# Targeting lymphatic vessels enhances bone regeneration by augmenting osteoclast activity in mouse models of amputation

**DOI:** 10.1172/JCI191906

**Published:** 2026-02-02

**Authors:** Neda Vishlaghi, Trisha K. Ghotra, Monisha Mittal, Ji Hae L. Choi, Sneha Korlakunta, Mingquan Yan, Janna L. Crossley, Danielle Griswold-Wheeler, Elnaz Ghotbi, Conan Juan, Shiri Gur-Cohen, Babak Mehrara, David A. Brown, Michael T. Dellinger, Lindsay A. Dawson, Benjamin Levi

**Affiliations:** 1 Department of Surgery, University of Texas Southwestern Medical Center, Dallas, Texas, USA.; 2 Department of Veterinary Physiology and Pharmacology, Texas A&M University, College Station, Texas, USA.; 3 Division of Regenerative Medicine, UCSD, San Diego, California, USA.; 4 Memorial Hospital Research Laboratories, New York, New York, USA.; 5 Duke Cancer Institute, Durham, North Carolina, USA.

**Keywords:** Bone biology, Vascular biology, Osteoclast/osteoblast biology

## Abstract

Although mammals generally demonstrate limited regenerative capacity compared with amphibians, the digit tip retains remarkable regenerative potential, providing a useful model to study successful mammalian regeneration. This process involves coordinated immune cell activity, vascular remodeling, and tissue reconstruction, yet the molecular checkpoints controlling regenerative versus fibrotic outcomes remain poorly understood. In mammals, regeneration of the digit tip (P3) proceeds through myeloid cell migration, early osteoclast-mediated osteolysis of the distal bone, and subsequent blastema-mediated regeneration. Here we test the hypothesis that lymphatic vessels regulate regenerative capacity by modulating local immune cell dynamics and osteoclast function. Using a lymphatic system–specific reporter line, we discovered that lymphatic vessels grow toward the nail region from the ventral side of the digit during quiescence and after amputation. These lymphatics closely surround, but do not invade, the native or regenerated bone. Unexpectedly, genetic, pharmacological, and surgical inhibition of lymphangiogenesis accelerated early osteolysis through enhanced transition of myeloid cells to osteoclasts, resulting in faster and more robust regeneration. These findings reveal a mechanism linking lymphatic vessel, immune regulation, and bone remodeling, suggesting that targeted manipulation of lymphatics dynamics may enhance regenerative outcomes after musculoskeletal injury.

## Abstract 2

Mammals have limited regeneration, restricted to the digit tip. Researchers found, reducing lymphatic vessels accelerates bone breakdown and enhances regeneration, revealing a potential therapeutic target.

## Graphical abstract

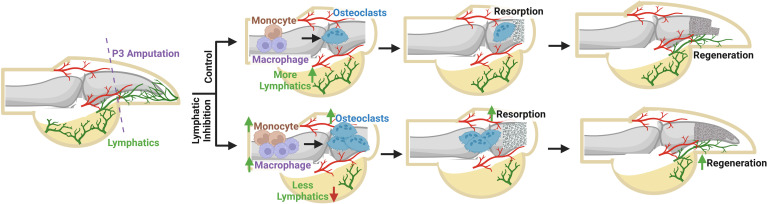

## Introduction

Tissue regeneration — the complete restoration of lost structures after injury — varies dramatically across species (1–3). While some animals can regenerate entire limbs, mammals typically heal through scarring, with one notable exception: the fingertip. Both mice and humans can regenerate the distal portion of the terminal phalanx (P3) but fail to regenerate more proximal injuries (4–7). This spatial restriction creates a unique experimental model — amputation at different levels of the same digit produces either regeneration (distal P3) or scarring (distal P2), allowing direct comparison of these healing outcomes (4, 8–18).

The regenerative response follows a defined sequence: Initial inflammation triggers osteoclast-mediated bone breakdown (osteolysis), which releases signals promoting blastema formation and ultimately new bone growth through intramembranous ossification (12, 19, 20). This process requires precise regulation of immune responses, vascular remodeling, and stem cell activation, serving as a model for successful mammalian regeneration. Although angiogenesis and osteogenesis are well studied, the roles of lymphangiogenesis and osteolysis remain largely unexplored.

The early phase of digit regeneration involves extensive tissue remodeling, with osteolysis playing a key role. Following P3 amputation, osteoclast activity increases to resorb existing bone, creating space for mesenchymal progenitors to form a blastema and regenerate new bone (21–23). The blastema, a hallmark of regeneration, is an undifferentiated cell mass that drives reconstruction of lost bone, skin, and soft tissue (4, 5, 12, 24–28). Importantly, blastema size is positively correlated with the extent of osteolysis (20). More extensive osteolysis results in a larger blastema, while reduced osteolysis leads to a smaller blastema (29). The link between osteolysis and regeneration suggests that early tissue breakdown may drive regenerative capacity. In contrast, osteoclast activity is reduced in non-regenerative P2 amputations compared with the robust response seen in regenerative P3 digits, suggesting that diminished osteoclast function may underlie failed regeneration in P2 (11).

The vascular environment represents a key distinction between regenerative and non-regenerative healing. In regenerative digit blastema, endothelial cell numbers are reduced, and oxygen tension fluctuates, including a transient hypoxic phase essential for regeneration (12, 30–32). Similarly, salamander limb regeneration exhibits reduced vascularity, suggesting the importance of a temporary avascular phase (33, 34). In contrast, non-regenerative mammalian wound healing involves rapid angiogenesis and dense granulation tissue (35).

Although blood vessel remodeling has been well studied, lymphatic vascularization, driven by the sprouting and migration of lymphatic endothelial cells (LECs), remains poorly understood (36). Lymphatic vessels regulate immune cell trafficking and tissue homeostasis through VEGF-C, VEGF-D, and their receptor VEGFR3, which promote LEC proliferation, migration, and tube formation (37–40). Recent studies have reported conflicting findings regarding the presence of lymphatics in bone during homeostasis and after injury (41–48). However, no studies have defined the spatial and temporal distribution of lymphatics in the uninjured or regenerating digit tip. Beyond fluid homeostasis, lymphatic vessels regulate immune cell dynamics (49, 50). Myeloid cells coordinate inflammation and regeneration via signaling pathways that restore tissue (29). Osteoclasts, derived from this lineage, are modulated by immune trafficking and signaling (26, 27)

Myeloid cells are essential for regeneration, as macrophage depletion in fracture and P3 amputation models reduce osteolysis and impair bone repair (20, 51–53). While enhancing early osteolysis has rarely been tested, loss of lymphatic drainage causes F4/80^+^ macrophage accumulation and inflammation-driven bone loss (54). Conversely, VEGF-C overexpression expands collecting lymphatics, improves drainage, and limits alveolar bone loss (45). How lymphatics regulate myeloid and osteoclast populations, and when lymphangiogenesis can be targeted to enhance digit regeneration, remains unknown.

In this study, we tested the hypothesis that lymphatic vessels regulate regenerative outcomes by modulating immune cell dynamics and osteoclast function. Using *Prox1-eGFP* reporter mice (55, 56), we mapped lymphatic vessel formation after digit amputation and found ventral expansion both before and throughout regeneration. Contrary to reports of lymphatics within bone (46), our data showed vessels surrounding but not penetrating bone. Genetic, pharmacologic, and surgical inhibition of lymphangiogenesis increased RANKL^+^ T cells and monocyte-to-osteoclast transition, enhancing early osteolysis and accelerating regeneration. These results reveal a lymphatic/myeloid/osteoclast axis and identify strategies to enhance digit regeneration.

## Results

### Lymphatic vessels maintain strict anatomical boundaries during quiescence and regeneration.

**Figure 1:**
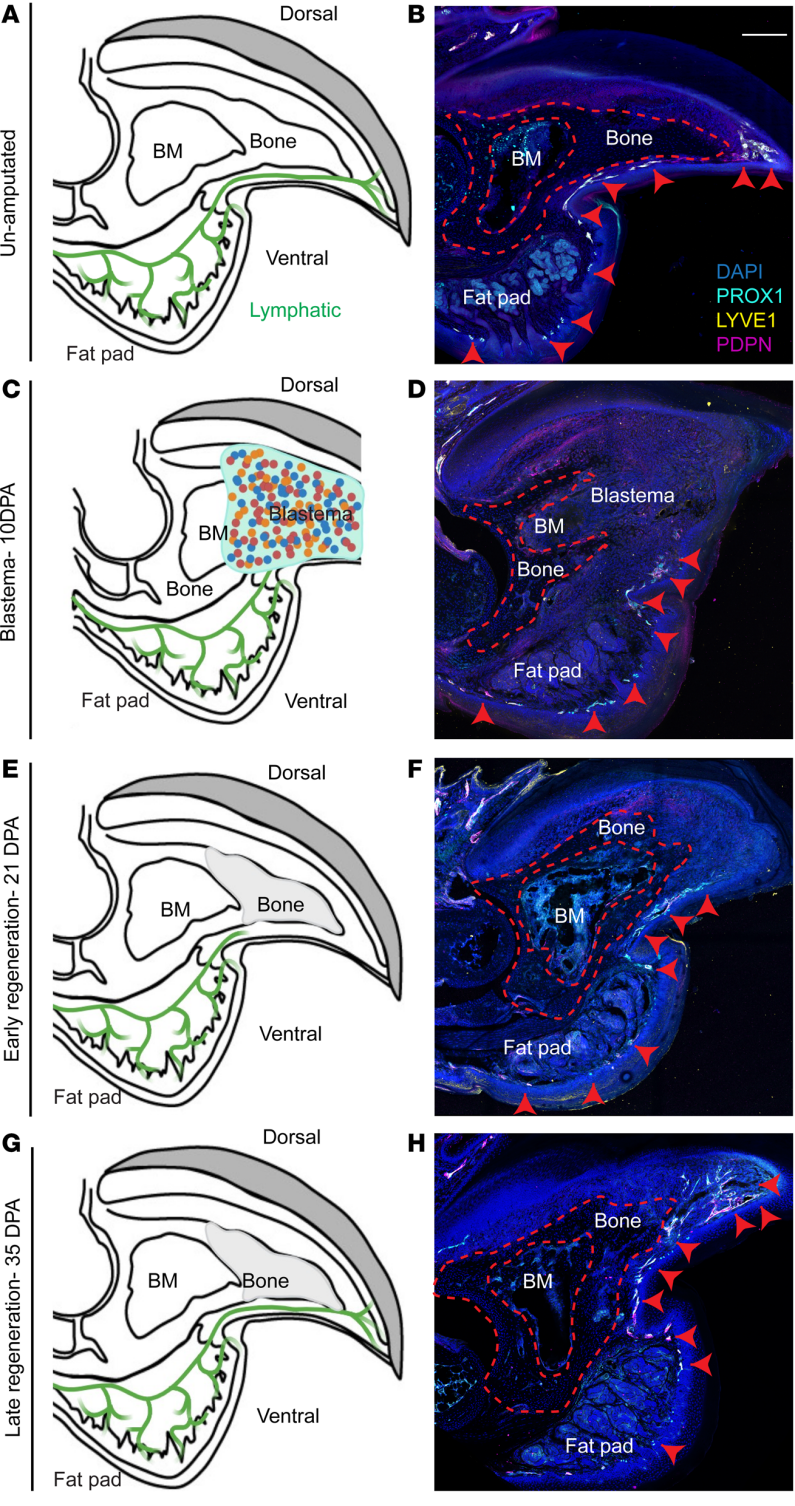
Lymphangiogenesis dynamics during digit tip regeneration. (**A**) Schematic of lymphatic vessels in an unamputated digit, showing the normal ventral lymphatic network. (**B**) Immunofluorescence staining for lymphatic markers LYVE1 (shown in yellow) and PDPN (shown in magenta) in *Prox1-eGFP* reporter mice demonstrates lymphatic vessels localized to the ventral side of the unamputated digit. Scale bar: 200 μm. (**C**) Schematic showing lymphatic remodeling during the blastema stage 10 DPA. (**D**) Immunofluorescence at 10 DPA reveals expansion of lymphatic vessels toward the forming blastema. (**E**) Schematic of the early regenerative stage (21 DPA). (**F**) Immunofluorescence at 21 DPA shows persistent ventral lymphatics closely associated with regenerating tissue. (**G**) Schematic of late regeneration (35 DPA). (**H**) Immunofluorescence at 35 DPA demonstrates reestablishment of a ventral lymphatic architecture resembling the unamputated state. BM, bone marrow; DPA, days postamputation.

To investigate lymphatic vessel dynamics during digit regeneration, we first examined lymphatic orientation in the P3 digit tip using *Prox1-eGFP* reporter mice. To validate lymphatic identity, we immunostained uninjured digits for LYVE1 (lymphatic vessel endothelial hyaluronan receptor 1) and PDPN (podoplanin), confirming triple-positive (GFP^+^LYVE1^+^PDPN^+^) lymphatic vessels (55–57). Lymphatic expansion was confined to the ventral side, extending along the P3 bone and wrapping around the tip (Figure 1, A and B). While closely associated with bone, no lymphatic vessels were detected within the P3 bone itself (Figure 1, A and B, and Supplemental Figure 1, B and J; supplemental material available online with this article; https://doi.org/10.1172/JCI191906DS1).

### Ventral restriction of lymphatic vessels persists throughout regeneration.

Recent studies suggest that dorsal-ventral patterning is not consistently preserved during digit regeneration (58–61). To determine whether lymphatics retain ventral localization, we performed P3 amputations in *Prox1-eGFP* reporter mice and co-stained for LYVE1 and PDPN at key regenerative stages. Lymphatic vessels remained confined to the ventral side of the digit, extending to the bone tip during regeneration (Figure 1, C–H, and Supplemental Figure 1, C–I).

Temporal analysis revealed distinct lymphatic distribution patterns during regeneration. At the wound-healing stage (3–7 days postamputation [DPA]), lymphatics localized mainly to the ventral fat pad and the P3 and fat pad interface (Supplemental Figure 1, C and D). This ventral pattern persisted through blastema formation (10–14 DPA) and early bone regeneration (21–28 DPA) (Figure 1, C–F, and Supplemental Figure 1, E–H). By late regeneration (35 DPA), lymphatics extended to the tip of the regenerated P3 bone, resembling uninjured digits (Figure 1, G and H, and Supplemental Figure 1I). Cross sections confirmed ventral localization and absence of lymphatics within cortical bone, consistent with prior reports (Supplemental Figure 1J) (41–45, 47, 48).

### Enhanced osteoclast activity distinguishes regenerative P3 from non-regenerative P2 digits.

**Figure 2:**
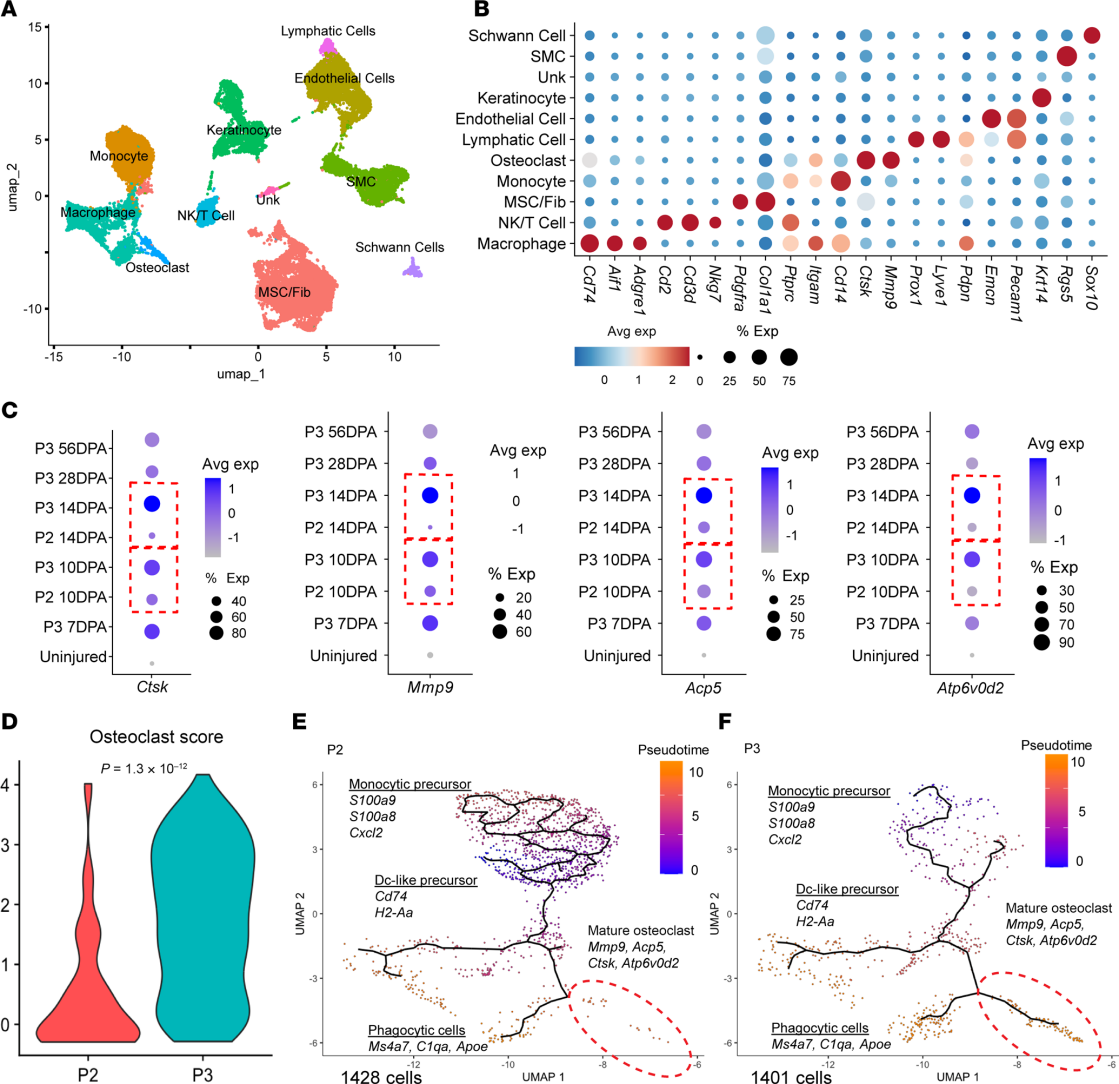
scRNA-Seq analysis reveals higher osteoclast gene expression in P3 compared with P2 amputated digits. (**A**) Uniform manifold approximation and projection (UMAP) showing cell clusters within P3 and P2. (**B**) Dot plot representation illustrates the expression of key marker genes for each unique cluster. (**C**) Dot plot representation illustrates the expression levels of key osteoclast marker genes at various time points postamputation, comparing between P2 and P3 amputation, which shows higher gene expression within P3 vs. P2 (dotted red box). (**D**) Violin plot showing osteoclast score comparing P2 and P3. (**E**) UMAP visualization of cell trajectory in P2 amputated samples. (**F**) UMAP visualization of cell trajectory in P3 amputated samples.

We first performed gene expression analysis using a published scRNA-Seq dataset (GSE135985) to compare fibrotic P2 and regenerative P3 amputation at 10 and 14 DPA (Figure 2, A and B). Given that lymphatics regulate myeloid cell trafficking, we hypothesized that differences in myeloid cell fate contribute to divergent healing outcomes. We therefore analyzed the expression of genes associated with myeloid cell fate after fibrotic P2 and regenerative P3 amputations. Several studies have identified specific signaling molecules that influence myeloid cells, osteoclast activity, and tissue remodeling, indicating that osteoclasts are essential for bone resorption, a crucial step in remodeling and regeneration (12, 25, 29).

Our comparative analysis revealed striking differences in osteoclast-related gene expression between P2 and P3 amputation. scRNA-Seq analysis of osteoclastogenesis markers in myeloid cells revealed that the P3 amputation had significantly higher expression levels of major osteoclast markers such as *Ctsk*, *Mmp9*, *Acp5*, and *Atp6v0d2* compared with P2 amputation during regeneration (Figure 2C). We calculated the osteoclast score using the average expression levels of selected genes (Supplemental Table 1), which revealed a significantly higher osteoclast signature in P3 tissue compared with P2 (Figure 2D).

To examine osteoclast differentiation dynamics, we performed cell trajectory analysis, which organizes cells along a pseudo-temporal axis within monocyte, macrophage, and osteoclast cell clusters. Data revealed clear progression toward mature osteoclasts in P3 compared with P2. The trajectory highlights active cell state transitions in the regenerative microenvironment, from monocytes to osteoclasts in P3 amputation (Figure 2, E and F, and Supplemental Figure 2, A and B). To validate these transcriptional findings, we performed tartrate-resistant acid phosphatase (TRAP) staining around 8 DPA, which showed more TRAP^+^ cells in P3 than in P2 (Supplemental Figure 2, C and D). Collectively, these data demonstrate that higher osteoclast activity may be critical for digit regeneration.

**Figure 3:**
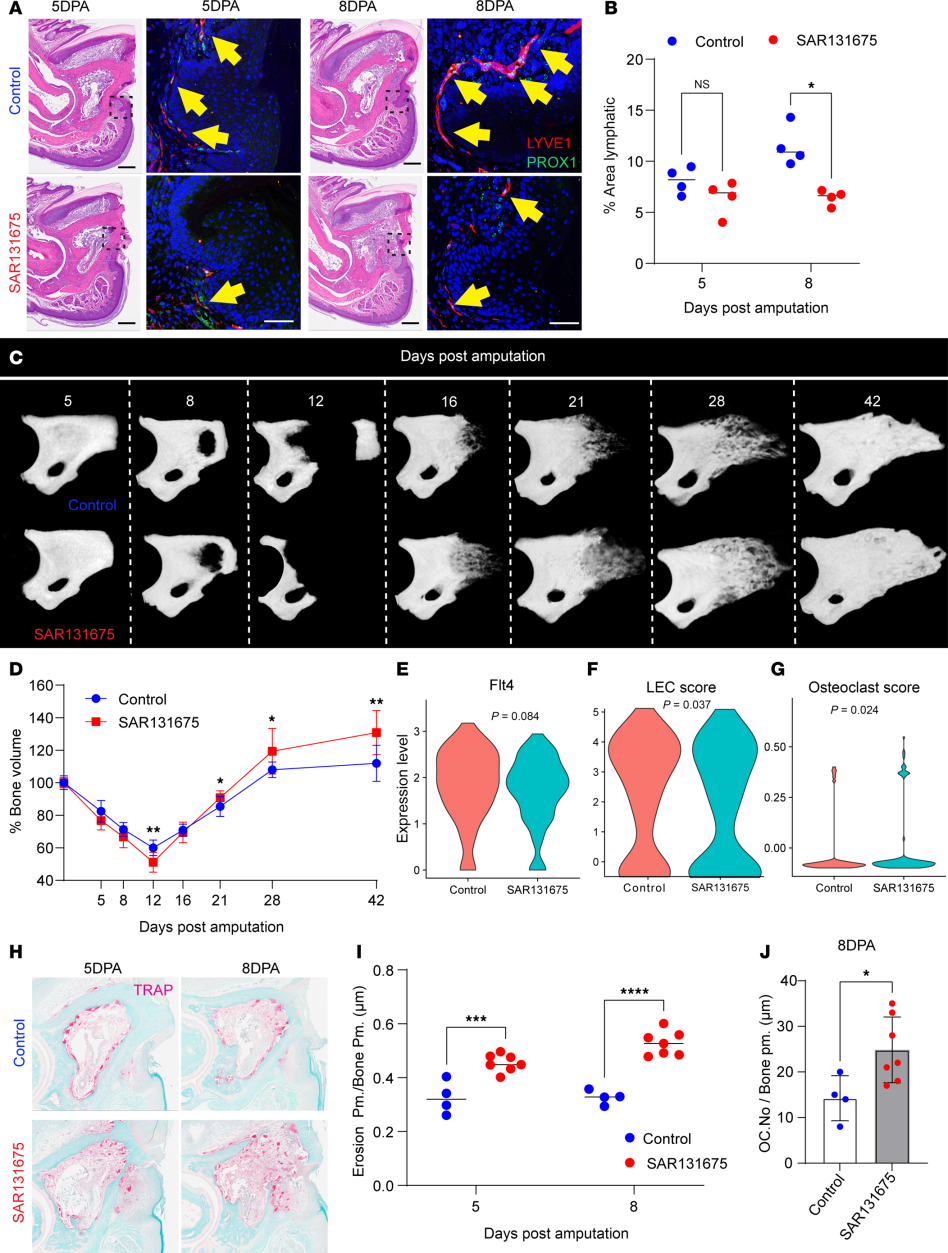
VEGFR3 inhibition enhances osteoclast-mediated bone resorption and accelerates bone remodeling during digit tip regeneration. (**A**) Immunofluorescence staining for lymphatic markers PROX1 (green) and LYVE1 (red) in control and SAR131675-treated digits at 5 and 8 DPA. Scale bars, 100 μm. (**B**) Quantification of lymphatic vessel area (PROX1^+^LYVE1^+^) shows significantly decreased lymphangiogenesis in SAR131675-treated digits at 8 DPA. Data are mean ± SD; Student’s *t* test; *P < 0.05. (**C**) Representative longitudinal micro-CT renderings of control (top) and SAR131675-treated (bottom) digits from 5 to 42 DPA show accelerated histolysis followed by enhanced bone regeneration in SAR131675-treated samples. (**D**) Quantification of bone volume over time demonstrates greater initial bone resorption and earlier regeneration in the SAR131675-treated group (*n* = 4–12 digits per group; mean ± SD; *P < 0.05, **P < 0.01). (**E**–**G**) Violin plots comparing (**E**) *Flt4* (VEGFR3) expression, (**F**) LEC score, and (**G**) osteoclast gene score between control and SAR131675-treated digits. VEGFR3 inhibition reduced lymphatic gene expression while increasing osteoclast-associated signatures. (**H**) TRAP-stained sections of distal P3 bone at 5 and 8 DPA show markedly elevated osteoclast activity (pink) in SAR131675-treated digits compared with controls. (**I**) Quantification of erosion perimeter/bone perimeter (μm) reveals significantly increased bone erosion in SAR131675-treated digits at 5 and 8 DPA. (**J**) Osteoclast number per bone perimeter (μm) is significantly higher in the SAR131675-treated group at 8 DPA. Data are mean ± SD; Student’s *t* test; *P < 0.05, ***P < 0.001, ****P < 0.0001.

*Pharmacologic VEGFR3 inhibition reduces lymphatic vessel formation*. Based on our observation that increased osteoclastogenesis correlates with enhanced regeneration, we next tested whether we could enhance P3 regeneration by further augmenting the osteolysis phase. Since VEGFR3 is one of the key receptors necessary for lymphangiogenesis, we first implemented pharmacologic VEGFR3 inhibition by dietary administering of SAR131675, 30 mg/kg, food (62–64). Immunofluorescence staining for the lymphatic markers PROX1 and LYVE1 was performed to identify double-positive lymphatic vessels (Figure 3A). The quantification revealed fewer lymphatic vessels in the SAR131675-treated group at both 5 DPA (*P* = 0.1657) and 8 DPA (*P* = 0.0031) (Figure 3B). CD31 staining showed no significant difference in blood vessel density between control and SAR131675-treated groups at 5 DPA (Supplemental Figure 3, A and B).

### VEGFR3 inhibition accelerates osteolysis and enhances regeneration.

Having confirmed that SAR131675 effectively reduced lymphatic vessels, we next assessed all phases of P3 digit regeneration by micro-CT (micro-computed tomography) (Figure 3C). At 5 DPA, there was no significant difference in bone volume between the groups (Figure 3, C and D; *P* = 0.0583). However, by 8 DPA, SAR131675-treated samples exhibited an enhanced osteolytic phase resulting in slightly reduced bone volume compared with vehicle controls (Figure 3, C and D; *P* = 0.1047). This trend of enhanced osteolysis persisted until 12 DPA, when we observed a significant decrease in bone volume (*P* = 0.0029) (Figure 3, C and D).

Importantly, this enhanced early osteolysis was followed by more robust regeneration. By 21 DPA (*P* = 0.0328), higher bone volume was observed in the treated group, suggesting a resolution of the enhanced osteolytic phase and the onset of enhanced bone formation (Figure 3, C and D). This enhanced regenerative response continued, with increased bone volume observed at 28 DPA (*P* = 0.0384) and 42 DPA (Figure 3, C and D; *P* = 0.0042). Using Masson’s trichrome staining, we confirmed our micro-CT finding that SAR131675 treatment increased osteoid formation (*P* = 0.0004) (Supplemental Figure 3, C and D). These data suggest that blocking VEGFR3 expedites the osteolysis phase as well as the overall regenerative process.

To understand the cellular mechanisms underlying enhanced regeneration, we performed scRNA-Seq on digit tissues treated with SAR131675 compared with controls (Supplemental Figure 3, E and F). Our data verified a lower expression of the *Flt4* gene within the SAR131675-treated group (Figure 3E), along with reduced expression of lymphatic-associated genes and a lower lymphatic score (65, 66) which confirms the presence of fewer LECs in the tissue following SAR131675 treatment (Figure 3F and Supplemental Table 1). Moreover, the expression levels of genes associated with osteoclastogenesis within myeloid cell clusters were elevated following VEGFR3 inhibition (67, 68) (Figure 3G and Supplemental Table 1). These findings suggest that VEGFR3 inhibition decreases lymphatic-related gene expression and shifts the microenvironment to favor osteoclastogenesis.

To validate that enhanced osteolysis resulted from increased osteoclast activity, we performed TRAP staining on P3 bone sections at critical osteolysis time points: 5 and 8 DPA (Figure 3H). Quantitative analysis confirmed significantly higher bone erosion at 5 DPA (*P* = 0.0005) and 8 DPA (*P* < 0.0001) (Figure 3I) and an increase in osteoclast numbers (*P* = 0.0291) (Figure 3J). These results indicate that therapeutic inhibition of VEGFR3 during P3 regeneration enhances early osteolysis and promotes bone regeneration.

### VEGFR3 inhibition alters immune cell dynamics and promotes myeloid cell osteoclast differentiation.

**Figure 4:**
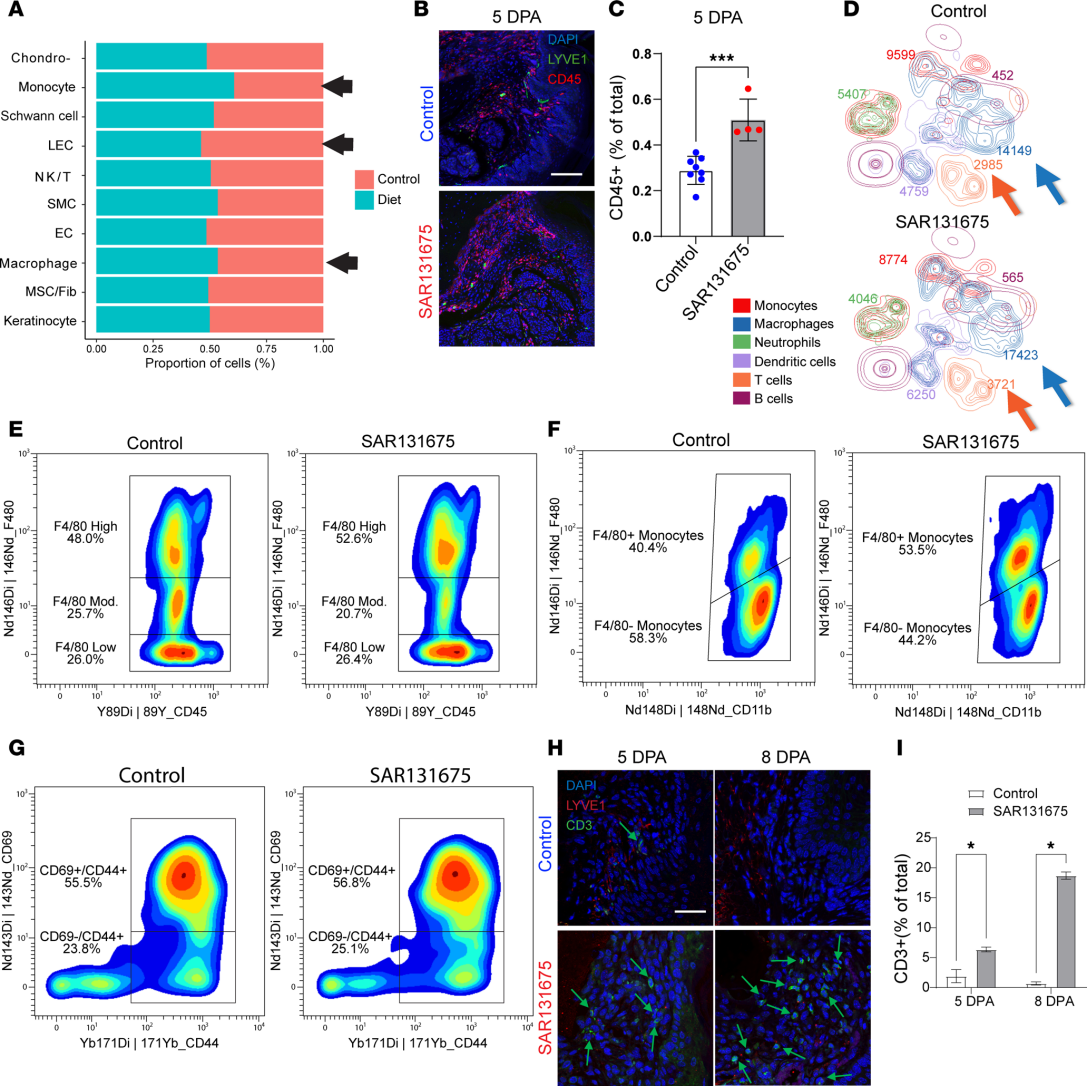
VEGFR3 inhibition alters myeloid and T cell composition and activation during digit tip regeneration. (**A**) Proportional cell distribution derived from scRNA-Seq showing increased representation of macrophage and monocyte populations, while shows decrease in LEC population in SAR131675-treated samples at 5 DPA. (**B**) Immunofluorescent staining for CD45 (red) and LYVE1 (green) in control and SAR131675-treated digits at 5 DPA. Scale bar: 100 μm. (**C**) Quantification of CD45^+^ cells shows a significant increase in immune cell infiltration in VEGFR3-inhibited digits at 5 DPA (mean ± SD; ***P < 0.001; Student’s *t* test). (**D**) CyTOF analysis of immune cells from control and SAR131675-treated mice demonstrates expanded myeloid cell clusters (blue and orange arrows) under VEGFR3 inhibition. (**E** and **F**) Contour plots of F4/80^+^ cells show elevated frequencies of F4/80-high myeloid cells (**E**) and increased F4/80^+^ monocyte subsets (**F**) in SAR131675-treated mice compared with controls. (**G**) Contour plots of CD69^+^CD44^+^ T cells in SAR131675-treated mice compared with controls. (**H**) Immunofluorescence staining for CD3 (green) and LYVE1 (red) at 5 and 8 DPA confirms elevated T cell infiltration in SAR131675-treated digits. Scale bar: 50 μm. (**I**) Quantification of CD3^+^ cells at 5 and 8 DPA shows a significant increase in the VEGFR3-inhibited group (*P < 0.05; mean ± SD; Student’s *t* test).

To elucidate how reduced lymphatic function enhances osteoclastogenesis, we next characterized the myeloid cell populations following digit amputation treated with SAR131675. We analyzed changes in cell numbers using scRNA-Seq data from SAR131675- treated and control samples at 8 DPA. Our analysis verified that there was a reduced number of LECs in the VEGFR3 inhibitor–treated group; however, there was an increased number of monocytes and macrophages following VEGFR3 inhibition (Figure 4A, black arrows).

To validate these transcriptional findings at the protein level, we stained P3 samples for the CD45 marker at 5 DPA and observed a significant increase in CD45^+^ cells in the SAR131675-treated group (*P* = 0.0005) (Figure 4, B and C).

To comprehensively characterize changes in immune cell populations, we employed CyTOF (cytometry by time-of-flight) at 5 DPA to phenotype myeloid cells. Gating for CD45^+^ cells verified an increased number of CD45^+^ cells in the diet-treated group (CD45^+^ cell numbers: control: 37,351; SAR131675: 40,779), consistent with our immunofluorescence data. We used several major markers to classify different cell populations, including monocytes, macrophages, neutrophils, dendritic cells, T cells, and B cells (Supplemental Figure 4A).

One striking difference between control and SAR131675-treated groups was an increase in F4/80^+^ macrophages in SAR131675-treated mice (Figure 4D, blue arrow). The proportion of high-expressing F4/80 cells was also increased in the SAR131675-treated group (Figure 4E). Furthermore, we separated Ly6C^+^CD11b^+^ monocytes into 2 groups based on F4/80 expression and observed an increase in F4/80^+^ monocytes along with a decrease in F4/80^–^ monocytes in the SAR131675-treated group (Supplemental Figure 4B). Further analysis confirmed the proportion of high-expressing F4/80 monocytes also increased in the SAR131675-treated group (Figure 4F). Recent studies identified CD45^+^CD11b^+^F4/80^+^CD206^+^ M2-like macrophages in the synovium of collagen-induced arthritis mice, which can differentiate into osteoclasts upon RANKL and M-CSF stimulation, acquiring osteoclast markers and bone-resorptive function both in vitro and in vivo (69).

Given that RANKL is crucial for monocyte-to-osteoclast differentiation, we next examined which cells were the primary source of RANKL. Utilizing an scRNA-Seq dataset of the digit tip at 8 DPA during the osteolysis phase, we found that CD3^+^ T cells were the primary cells expressing RANKL (*Tnfsf11*) within the digit tissue (Supplemental Figure 4C). We further investigated the changes within T cell populations impacted by VEGFR3 inhibition using CyTOF. Our analysis showed that the number of CD3^+^ T cells increased within the SAR131675-treated group (CD3^+^ cells: control: 2,985; SAR131675: 3,721) (Figure 4D, orange arrow), and the proportion of activated T cells (those known to express *Tnfsf11*) (70–72) also increased after VEGFR3 inhibition (CD44^+^CD69^+^ high: control: 55.5%; SAR131675: 56.8%) (Figure 4G). CD3 staining at 5 and 8 DPA confirmed a significant increase in CD3^+^ T cells in the SAR131675-treated group compared with controls (*P* < 0.002) (Figure 4, H and I). Together, these findings suggest that VEGFR3 inhibition increases the number of F4/80^+^ macrophages and monocytes within the digit microenvironment, accompanied by a higher number of RANKL-expressing T cells, thereby promoting the early osteoclastogenesis phase necessary for eventual digit regeneration.

### Genetic ablation of lymphatic vessels confirms their regulatory role in regeneration.

**Figure 5:**
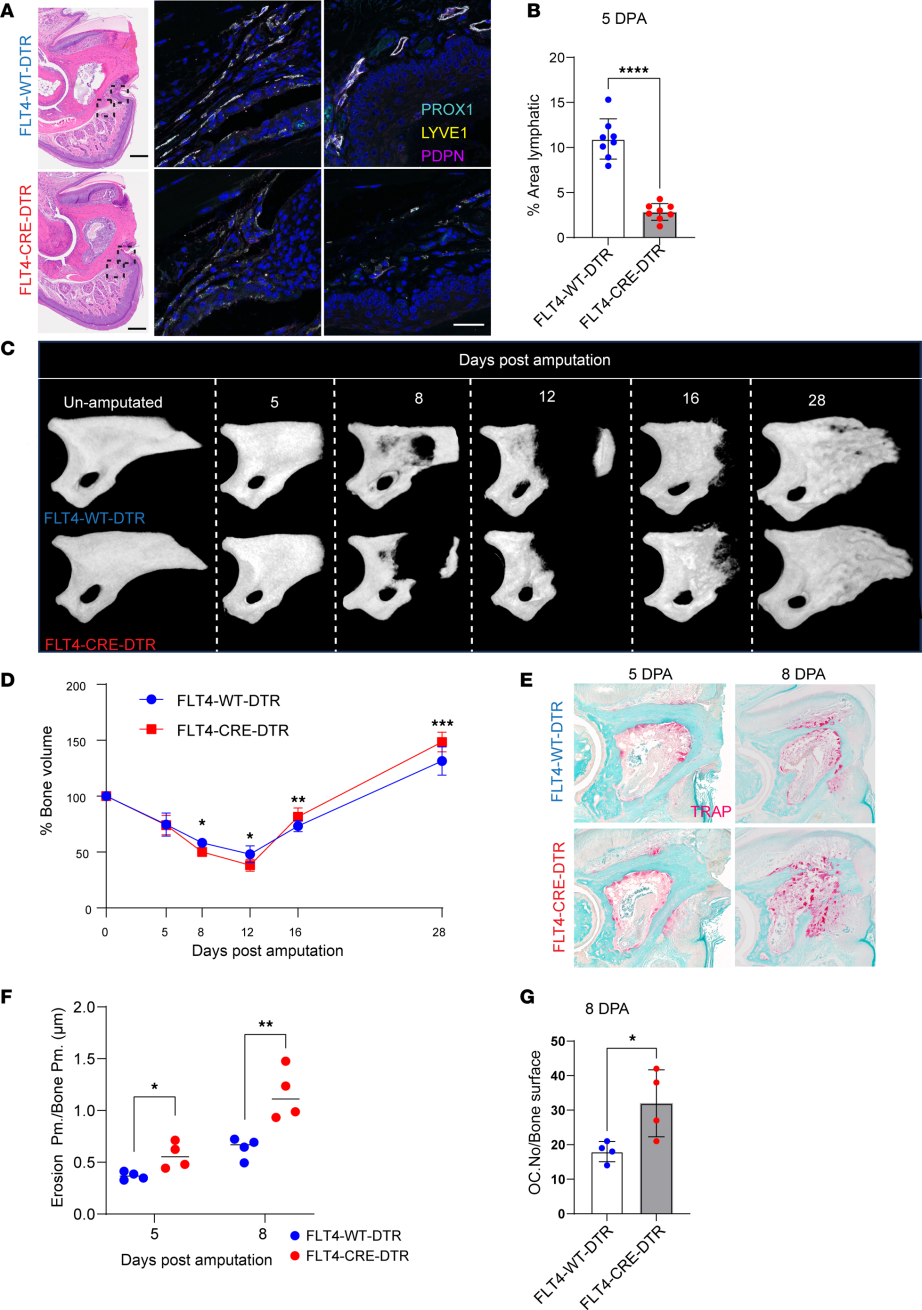
Impact of LEC ablation on osteoclast activity and digit tip regeneration. (**A**) Immunofluorescent staining for lymphatic markers (PROX1, LYVE1, and PDPN) in control and *Flt4Cre^ERT2+^ iDTR^+^* mice shows fewer lymphatic vessels formed during regeneration in *Flt4Cre^ERT2+^ iDTR^+^*. (**B**) Quantification of lymphatic area within the tissue shows significant differences between the control and *Flt4Cre^ERT2+^ iDTR^+^* group at 5 DPA. (**C**) Representative micro-CT renderings of control digits (top) and a *Flt4Cre^ERT2+^ iDTR^+^* (bottom) 5, 8, 12, 16, and 28 DPA. (**D**) Quantification of bone volume at 5, 8, 12, 16, and 28 DPA. (*n* = 4–12 digits/group.) (**E**) Histology images of amputated P3 bone stained with TRAP at 5 and 8 DPA comparing control and *Flt4Cre^ERT2+^ iDTR^+^* group. (**F**) Comparison of osteoclast-mediated bone erosion at 2 major osteolysis time points (5 and 8 DPA). (**G**) Comparison of osteoclast numbers at major osteolysis time point (8 DPA). Significance was determined using Student’s *t* test, with data presented as mean ± SD (*P < 0.05; ***P < 0.005; ****P < 0.0001).

To genetically validate our pharmacological findings, we used *Flt4Cre^ERT2^ iDTR* mouse line (61), to ablate *Vegfr3*-expressing cells during digit regeneration. Diphtheria toxin was administered into the footpads for 3 consecutive days, starting 1 day before amputation, effectively eliminating *Flt4*^+^ lymphatic vessels at the injury site. Immunostaining confirmed effective lymphatic ablation, with 5 DPA tissues showing markedly reduced PROX1^+^LYVE1^+^PDPN^+^ vessels (Figure 5, A and B; *P* < 0.0001). CD31 staining showed no significant change in blood vessel area at 5 DPA (Supplemental Figure 5, A and B).

To evaluate the impact of genetic lymphatic ablation on regeneration, we conducted micro-CT analysis across key time points (Figure 5C). Quantification showed enhanced P3 bone regeneration in lymphatic-ablated (*Flt4Cre*^ERT2+^ *iDTR*^+^) mice compared with controls, mirroring pharmacologic results. Bone volume was similar at 5 DPA (*P* = 0.94) but reduced at 8 DPA due to increased osteolysis (*P* = 0.015) (Figure 5D). Enhanced osteolysis persisted through early regeneration, with *Flt4Cre*^ERT2+^ *iDTR*^+^ mice showing lower P3 volume at 12 DPA (*P* = 0.0335) (Figure 5D), suggesting accelerated bone resorption similar to VEGFR3 inhibition. By 16 DPA, bone volume increased significantly (*P* = 0.0081), and by 28 DPA, mutants showed greater regeneration (*P* = 0.0005), indicating faster histolysis followed by enhanced bone formation (Figure 5, C and D). Masson’s trichrome staining showed increased osteoid formation in *Flt4Cre*^ERT2+^ *iDTR*^+^ digits at 12 DPA (Supplemental Figure 5, C and D). TRAP staining confirmed greater bone erosion (5 DPA, *P* = 0.024; 8 DPA, *P* = 0.009) and more osteoclast cells (8 DPA, *P* = 0.033) (Figure 5, E–G). These results parallel pharmacologic findings (Figure 3), showing that LEC ablation accelerates osteolysis and enhances P3 regeneration through reduced VEGFR3 signaling. Together, our data showed that reduced VEGFR3 signaling and lymphatic vessel number promote early osteolysis and improved P3 regeneration.

### Constitutive Vegfr3 deficiency confirms lymphatic regulation of regeneration.

To further validate our findings, we used *Chy* (*Vegfr3^wt/mut^*) mice, which exhibit reduced lymphatic development (73–75). Previously used by our group (74) and others (76–78), this model enables investigation of how lymphatic hypoplasia influences digit regeneration. These mice carry a point mutation in the kinase domain of VEGFR3 that impairs kinase activity and exerts a dominant-negative effect on VEGFR3 signaling (47, 75). At 7 DPA, *Chy* digits showed reduced LYVE1^+^PDPN^+^ lymphatic vessels (Supplemental Figure 6A), with quantification confirming a significant decrease (*P* < 0.0001) (Supplemental Figure 6B). Micro-CT analysis showed that *Chy* mice exhibited enhanced digit regeneration (Supplemental Figure 6C). Quantification revealed increased P3 bone regeneration in mutants, with no significant differences at 7 DPA (*P* = 0.5153) or 10 DPA (*P* = 0.0849). By 14 DPA, mutants showed early new bone formation but lower bone volume (*P* = 0.0202), indicating enhanced osteolysis from lymphatic deficiency (Supplemental Figure 6, C and D). Following the enhanced osteolytic phase, *Chy* mutants exhibited markedly improved regenerative outcomes, with significantly increased bone volume at 21 DPA (*P* = 0.0432) and 28 DPA (*P* = 0.0004) (Supplemental Figure 6, C and D). This enhanced regeneration was further supported by trichrome staining, which revealed increased osteoid deposition at 14 DPA (Supplemental Figure 6, E and F).

To assess whether enhanced regeneration correlated with osteoclast activity, TRAP staining at 7 and 10 DPA revealed greater bone erosion and increased osteoclast numbers in *Chy* mice at 7 DPA (*P* = 0.0454), which normalized by 10 DPA (Supplemental Figure 6, G–I). As expected, *Chy* mice exhibited hind paw lymphedema due to impaired lymphatic drainage (Supplemental Figure 6J). These results reinforce that *Vegfr3*-mediated lymphatic function regulates early osteolysis and subsequent bone regeneration, consistent with our pharmacologic and ablation models.

### Surgical manipulation of lymphatics confirms their regulatory role in regeneration.

To validate our findings in a clinically relevant context, we performed surgical lymph node removal (LNR) on one limb, using the contralateral side as sham, to modulate lymphatic function (79).

LNR effectively reduced lymphatic vessels, confirmed by decreased LYVE1^+^PDPN^+^ area (*P* = 0.0023) and reduced LEC gene expression at 8 DPA (Supplemental Figure 7, G–I).

**Figure 6:**
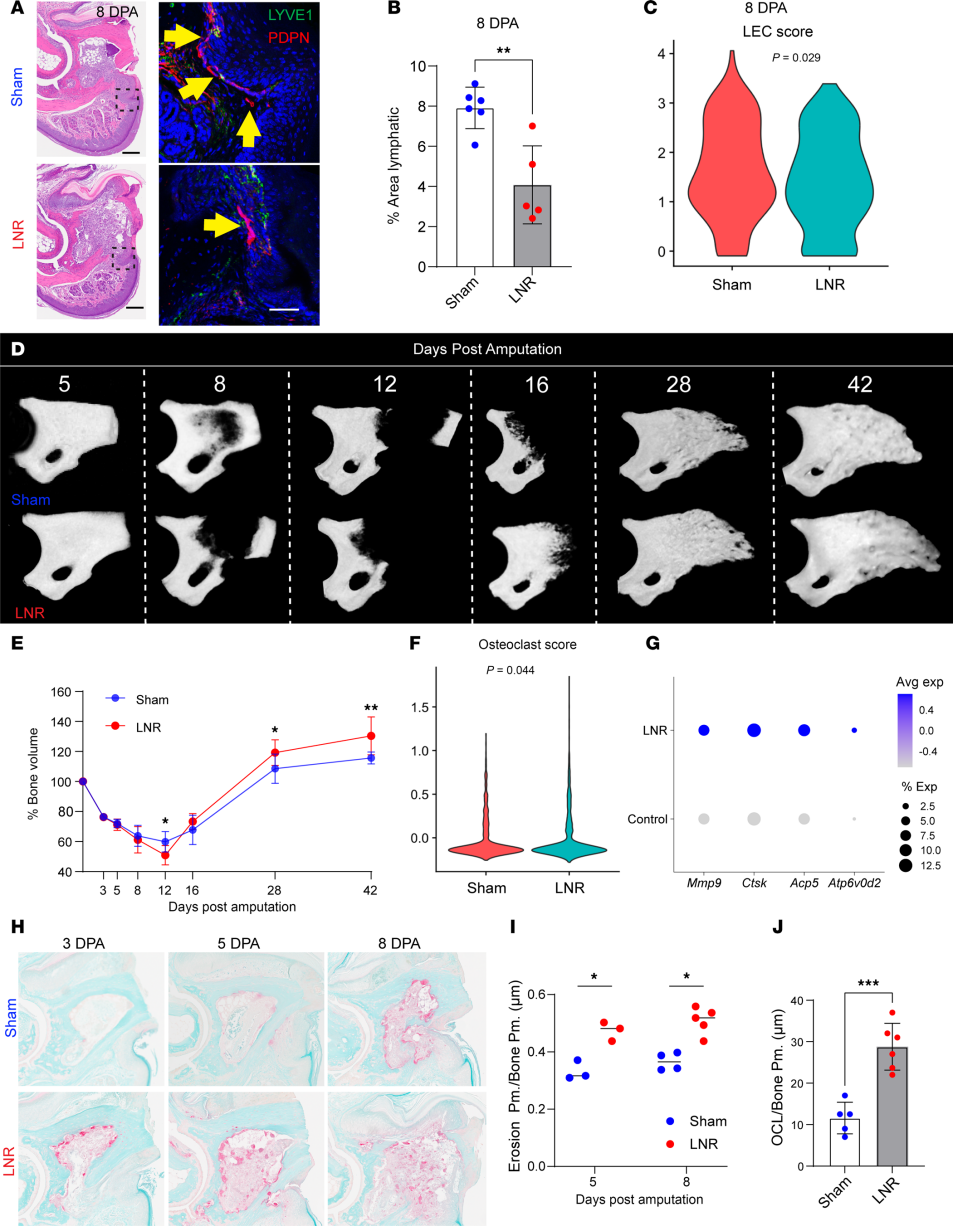
LNR enhances bone regeneration during digit tip repair. (**A**) Immunofluorescence staining for lymphatic markers (LYVE1 and PDPN) in sham and LNR digits at 8 DPA showing a marked reduction in lymphatic vessel density in LNR samples (yellow arrows). (**B**) Quantification of lymphatic area reveals a significant decrease in lymphatic coverage in LNR digits compared with sham controls (*P* < 0.01; mean ± SD, Student’s *t* test). (**C**) Violin plot showing reduced lymphatic endothelial cell (LEC) score in LNR samples relative to sham controls (*P* = 0.029). (**D**) Representative longitudinal micro-CT reconstructions of sham (top) and LNR (bottom) digits at 5, 8, 12, 16, 28, and 42 DPA. (**E**) Quantification of bone volume over time demonstrates accelerated bone regrowth in LNR digits (*P < 0.05, **P < 0.01; *n* = 4–12 digits/group). (**F**) Violin plot showing elevated osteoclast scores in LNR digits (*P* = 0.044). (**G**) Dot plot of osteoclast-related gene expression (*Mmp9*, *Ctsk*, *Acp5*, *Atp6v0d2*) showing higher expression in LNR versus control samples. (**H**) TRAP staining of P3 bones at 3, 5, and 8 DPA illustrating increased osteoclast activity in LNR digits. (**I**) Quantification of osteoclast-mediated bone erosion at 5 and 8 DPA (*P* < 0.05). (**J**) Quantification of osteoclast number at 8 DPA (**P < 0.001). Data are shown as mean ± SD, and significance was determined using Student’s *t* test.

Micro-CT imaging revealed accelerated osteolysis in the LNR group, with significantly lower P3 bone volume at 12 DPA (*P* = 0.017), followed by enhanced regeneration and increased bone volume at 28 DPA (*P* = 0.0353) and 42 DPA (*P* = 0.0072) (Figure 6, D and E).

Masson’s trichrome staining confirmed increased osteoid formation (*P* = 0.0004) (Supplemental Figure 7, D and E, black arrows). Consistent with genetic and pharmacologic models, LNR elevated osteoclastogenesis-related gene expression and scores (Figure 6, F and G) and increased osteoclast activity and bone erosion at 5 and 8 DPA (*P* = 0.0186; *P* = 0.0213) (Figure 6, H–J). Our scRNA-Seq analysis further confirmed upregulation of RANKL (*Tnfsf11*) expression within CD3^+^ T cells from LNR samples compared with the sham group, supporting our hypothesis that lymphatic modulation enhances osteoclastogenesis through immune cell regulation (Supplemental Figure 7F).

Together, these results demonstrate that LNR accelerates osteolysis and enhances digit bone regeneration, phenocopying VEGFR3 inhibition.

### Osteoclast activity is required for enhanced regeneration.

Having demonstrated enhanced regeneration through lymphatic reduction across 4 independent models (VEGFR3 inhibitor, *Flt4Cre^ERT2+^ iDTR^+^*, *Chy*, and surgical removal of lymph nodes), we examined the impact of inhibiting osteoclast activity. We treated mice with Zometa (zoledronic acid), a bisphosphonate that effectively disrupts both the formation and function of osteoclasts (80).

Using 3D renderings of micro-CT scans, we observed substantial and significant impairment in both the osteolysis phase (days 5–8) and overall digit regeneration (day 28) (Supplemental Figure 7, G–I). Quantitative analysis revealed reduced bone volume and length in Zometa-treated mice (Figure 6, K and L). These findings demonstrate the essential role of osteoclast-mediated bone degradation in normal bone regeneration. Zometa treatment disrupted the natural bone turnover process, leading to abnormal bone formation in the regenerating digit. Our results indicate that balanced bone resorption and formation are vital for successful tissue regeneration, suggesting that modulation of osteoclast activity could serve as a therapeutic target for optimizing regenerative outcomes.

## Discussion

Successful regeneration requires precise coordination between tissue breakdown, immune responses, and reconstruction. Here we identify lymphatic vessels as key regulators of this process, revealing that reduced lymphatic function enhances digit regeneration through modulation of myeloid cell dynamics and osteoclast activity. These findings have significant implications for regenerative medicine, as over 4.8 million hand and finger injuries are treated annually in emergency rooms, with over 45,000 finger amputations performed annually in the United States, accounting for two-thirds of all pediatric hand injuries (81–85).

Current therapeutic approaches have significant limitations. While regenerative medicine typically relies on the use of native or bioengineered scaffolds, stem cell therapy, or a combination of these approaches (86), these interventions rarely achieve full functional recovery.

Understanding endogenous regenerative mechanisms offers an alternative strategy. The mouse digit tip regeneration is an important preclinical model for studying human digit and limb epimorphosis (19, 87, 88). A key feature of this system is digit blastema, which creates its own scaffold (89) and coordinates the patterned regeneration of bone (90), blood vessels (32), and nerves (91). This endogenous regeneration process surpasses traditional regenerative medicine approaches like bioengineered scaffolds, osteoinductive signals, and autologous grafts.

Our study reveals a mechanism controlling regeneration through lymphatic vessel regulation of immune cell dynamics and osteoclast function. The P3 regenerative response differs fundamentally from typical wound healing. While regeneration is restricted to the distal tip of the third phalangeal element (P3), amputation of the second element (P2; proximal to the distal interphalangeal joint) lacks a targeted osteoclast-mediated osteolysis phase and results in bone truncation and soft tissue fibrosis (11, 19, 90, 92–100).

P3 amputation initiates robust bone regeneration within 42 days (90, 95, 97, 101, 102), starting with myeloid cell migration and osteoclast-mediated bone degradation, which releases regeneration signals and blastema cells. This osteolysis process is followed by wound closure and bone regeneration through intramembranous ossification, providing a model for studying osteoclast and osteoblast activity in vivo (11, 19, 90, 95–100). Nearly all previous digit-regenerative approaches and studies have focused on osteogenic progenitor cells while fewer studies showed the role of early postinjury osteoclastogenesis, which is necessary to clear the damaged bone and to release blastema cells and other cell signals. Prior work has demonstrated that depletion of osteoclasts mitigates the early osteolysis phase and inhibits bone repair and regeneration (20, 51–53).

Our work demonstrates that lymphatic vessels actively regulate regenerative capacity by modulating myeloid cell dynamics and osteoclast function. We demonstrated that osteoclast activity, which is crucial for bone remodeling, is more pronounced in P3 regenerative tissues. We found that the ablation of lymphatic vessels alters the tissue microenvironment, promoting osteoclastogenesis by increasing the presence of myeloid cells, such as macrophages and F4/80^+^ monocytes.

These findings align with and extend previous studies of regenerative mechanisms. Prior work has established that mouse digit tip regeneration involves early osteolysis and robust osteoclast activity, with osteoclasts derived from the monocyte/macrophage lineage (103). During early regeneration, osteoclasts are highly abundant and can cause substantial phalangeal resorption, reducing bone volume by nearly 50% (12, 31). Notably, digits injected with clodronate liposomes (Clo-Lipo), which deplete macrophages, exhibited a significant reduction in F4/80^+^ cells and osteoclast activity compared with PBS-Lipo controls, resulting in inhibited regeneration (29).

Notably, selective depletion of osteoclasts using free clodronate, which was purported to target osteoclasts without affecting macrophages, also led to impaired regeneration. Interestingly, the phenotype resulting from osteoclast depletion could be reversed by rapid reepithelialization, while the phenotype from concurrent macrophage and osteoclast depletion could not (29).

Our findings extend this work by revealing how lymphatic vessels regulate this process. We found that inhibition of VEGFR3 signaling, as well as ablation of *Vegfr3*-expressing LECs, increased both osteoclast numbers and bone resorption, along with a rise in macrophage numbers. Our analysis confirmed that increased bone resorption was associated with accelerated bone formation, as evidenced by osteoid formation.

To validate the importance of osteoclast activity, we used the osteoclast inhibitor, zoledronic acid, during the osteolysis phase and observed that, in the absence of osteolysis, the cortical bone structure of the P3 phalanx was unable to regenerate. Partial bone resorption and the emergence of newly formed bone occurred at a later stage and exhibited abnormal characteristics. This finding suggests that osteoclast activity is not confined to the early stages of regeneration but can be reactivated later, influencing the overall progression and outcome.

Our findings parallel recent observations in axolotl limb regeneration, where the use of the same osteoclast inhibitor led to defective tissue integration (104). The observed phenotypes in axolotl included angulation of the radius, heterotopic bone formation, and complete separation between mature and regenerated structures. The axolotl study suggests that a gradient of integration phenotypes correlates with the extent of tissue resorption, where greater resorption results in better integration, and that effective resorption is essential for successful skeletal regeneration (104). Similarly, our data demonstrate that increased osteoclast activity and bone resorption are necessary for bone regeneration. Future studies will investigate whether differences in myeloid cell migration and osteoclast fate contribute to failed fracture repair in patients with fracture non-union.

Our work also resolves a significant controversy regarding lymphatic localization in bone. Although recent studies have identified the central role of lymphatics in skin, cardiovascular, and gastrointestinal tissue repair and regeneration, previous analyses of human and mouse bones have revealed an absence of lymphatic vessels (41–45). Previous studies using tissue-clearing and 3D imaging demonstrated that bones do not contain lymphatics but instead are surrounded by lymphatic vessels (47, 48). However, authors of a recent study suggest that lymphatics do indeed exist within bone before and after injury (46).

Using multiple complementary approaches, including *Prox1-eGFP* reporter and co-stain for LYVE1 and PDPN, we demonstrate that lymphatics are restricted to the ventral side of the digit and do not invade the bone prior to injury. During the regenerative process, lymphatics maintain their position in the ventral region of the digit and remained outside of the cortical bone without osseous invasion at all stages, including at the final stage of digit regeneration.

These findings have important implications for regenerative biology. The extraskeletal localization and ventral restriction of lymphatic vessels align with recent studies emphasizing the role of dorsal–ventral patterning in digit regeneration (58–61). Future studies will employ tissue clearing to further validate these observations. Although macrophage depletion disrupts digit tip regeneration by impairing bone histolysis and wound closure (29), the role of lymphatics in regulating immune cell phenotype and inflammation during regeneration remains unexplored. Lymphatic vessels may influence regeneration indirectly by modulating immune cell trafficking at the wound site, thereby affecting osteoclast activity and bone resorption.

Several important questions remain regarding lymphatic regulation of regeneration. First, although *Chy* and *Flt4CreERT2^+^ iDTR^+^* models are well established for studying lymphedema, the limited lymphatic network within the mouse digit constrains functional analyses. Lymphedema was evident in *Chy* mice but absent in *Flt4CreERT2^+^ iDTR^+^*, LNR, and SAR131675-treated animals. Because *Flt4* is also expressed in bone marrow endothelium (105, 106) ablation in *Flt4CreERT2^+^ iDTR^+^* mice may affect marrow vasculature; although H&E staining revealed no morphological defects, the small marrow space of P3 restricts deeper functional assessment.

Second, the mechanisms by which lymphatic vessels influence osteoclast differentiation remain unresolved. Our findings suggest that lymphatic depletion increases myeloid-to-osteoclast transition, likely through impaired drainage, yet the scarcity of LECs in our scRNA-Seq data limited detailed interaction analysis.

Third, while we targeted osteoclast activity with Zometa early postamputation, micro-CT data revealed continued bone resorption after treatment ended, suggesting prolonged or rebound osteoclast activity, as seen in bisphosphonate-treated patients (107–109). Finally, key regenerative questions persist: How do inflammatory dynamics differ between non-regenerative P2 and regenerative P3 healing? What immune mechanisms determine fibrosis versus regeneration? Addressing these will clarify how immune-lymphatic interactions govern tissue repair.

## Methods

### Sex as a biological variable.

Both male and female mice were used for all transgenic experiments. For SAR131675 treatment, LNR, and Zometa experiments, only females were used to minimize cage aggression that could affect regeneration. Both our data and prior studies indicate that sex does not significantly influence digit tip–regenerative outcomes.

### Experimental model details.

Mice were housed 5 per cage with standard chow (Envigo-Teklad 2016), water, and a 12-hour light/12-hour dark cycle. For SAR131675 experiments, 30 mg/kg, 30 ppm SAR131675 was incorporated into chow starting 5 days before amputation and maintained throughout the study. *Chy* mice (male and female; PMID: 11592985) and *Prox1-eGFP* mice (male and female) (55) were used. Female C57BL/6J mice (The Jackson Laboratory) were used for SAR131675, LNR, and Zometa experiments. *Flt4Cre^ERT2^ iDTR* mice (110) were obtained from MSKCC. *Cre* recombination was induced with tamoxifen citrate chow (400 mg/kg, 1 week) and intraperitoneal (i.p.) tamoxifen (75 mg/kg, days 0 and 2), followed by a 4-day washout before injury. For iDTR activation, diphtheria toxin (10 μL; 5 ng) was injected into the hind paw fat pad daily for 3 days, starting 1 day before injury. In Zometa studies, mice received sterile saline (100 μL; i.p.) or zoledronic acid (1.2 μg/100 μL; i.p.) every other day beginning on the day of amputation.

### Digit amputation model.

Mice were anesthetized with isoflurane, and hair was shaved to expose the surgical site. The distal tip of the terminal phalanx (P3) on digits 2 and 4 of each hind limb was amputated using a sterile #11 scalpel under aseptic conditions. Under a dissection microscope, the hind paw was positioned to expose the medial surface, and the cut was made parallel to the footpad, transecting the nail organ, dermis, P3 bone, vasculature, and nerves, but sparing the marrow cavity and fat pad. The amputation removed approximately 15%–20% of the P3 bone volume.

### LNR.

Mice were anesthetized with isoflurane, and hair was shaved to expose the incision site. A 4 mm incision lateral to the midline (hind paw or posterior knee) exposed the inguinal and popliteal lymph nodes. Nodes were removed by blunt dissection, and incisions were closed with 5-0 Vicryl sutures. Buprenorphine ER (0.3 mg/kg, s.c.) was administered for analgesia.

### Histology and immunofluorescence staining.

Digits were fixed in 4% paraformaldehyde (PFA) for 24 hours at 4°C, washed with PBS, and decalcified for 3 weeks in 14% EDTA (pH 7.4). Samples were embedded in OCT, gelatin (20% sucrose, 2% PVP, 8% gelatin), or paraffin and sectioned longitudinally at 10–30 μm for immunofluorescence, H&E, or Masson’s trichrome staining. For IF, frozen sections were washed in 1× TBS-T (0.05% Tween-20), blocked for 2 hours (1% BSA, 2% donkey/goat serum, 0.05% Triton X-100, 300 mM glycine, pH 8.4), and incubated overnight at 4°C with primary antibodies: LYVE1 (R&D Systems AF2125, 1:100), PDPN (Abcam ab11936, 1:100), CD45 (Cell Signaling Technology [CST] D3F8Q, 1:100), CD31 (CST 77699, 1:100), CD3 (Abcam ab16669, 1:100), and PROX1 (Abcam ab101851, 1:100). After washing, slides were incubated with Alexa Fluor–conjugated secondary antibodies (Invitrogen A21447, A78961, and A21206; 1:500) for 2 hours, counterstained with Hoechst 33342, and mounted in ProLong Gold (Invitrogen P36980). Images were acquired using a Leica SP8 confocal microscope with 10× or 40× objectives, and H&E/trichrome slides were scanned using a Hamamatsu NanoZoomer 2.0-HT.

### TRAP staining.

For TRAP staining, samples were fixed in 4% PFA for 24 hours and decalcified with 14% EDTA for 2–3 weeks. The samples were dehydrated through an ethanol series, embedded in paraffin, and sectioned. After deparaffinization, 5 μm–thick sections were incubated in buffered Naphtol-AS-BI-phosphate/Fast Red Violet LB (pH 5.2) with 50 μM sodium tartrate (Takara Bio) for 4 hours at 37°C. Slides were counterstained with Fast Green, developed in running tap water for 10 minutes, dehydrated, and mounted.

### Micro-CT.

All harvested digits were imaged using VivaCT 80 (SCANCO Medical) with an image pixel resolution of 4.9 μm, 55 kVp energy, 145 μA intensity, and 300 ms exposure time. Zometa mouse digits were imaged using a Skyscan 1272 (Bruker) with image pixel resolution of 4.0, 40 kVp energy, 200 μA, and 1,800 ms exposure time. Image serial sections were stacked and exported as dicom files into Dragonfly ORS for analysis.

### Digit statistical analysis.

Bone volume and length were quantified in Dragonfly ORS by an operator following a blinded protocol. Regenerated P3 bone volume was segmented and normalized to day 0 values to calculate percentage regeneration. Statistical analyses were performed in GraphPad Prism using unpaired 2-tailed *t* tests or 1-way ANOVA, with *P* < 0.05 considered significant.

### Cell preparation for CyTOF and scRNA-Seq.

Digits from 10 mice were pooled per sample. Hind limb digits 2–4 were amputated at the proximal P2 level and bisected longitudinally to expose internal tissues. Single-cell suspensions were prepared as described previously (65). Briefly, tissues were digested in Collagenase I (3 mg/mL), Collagenase II (2 mg/mL), and Dispase (3 mg/mL) in DMEM for 45 minutes at 37°C with agitation (150 rpm). Digests were quenched with DMEM + 10% FBS, filtered (40 μm), and centrifuged (400*g*, 5 minutes, 4°C). Pellets were treated with ACK lysis buffer for 2 minutes to remove RBCs. Cell viability was determined using trypan blue on Countess III (Thermo Fisher Scientific), and only samples with more than 75% viability were processed for sequencing.

### CyTOF analysis.

FCS files from SAR131675-treated and control samples were analyzed in OMIQ (Dotmatics). Data were gated to exclude debris, doublets, and dead cells based on Iridium signal and event length. CD45^+^ live single cells were subsampled, and major immune populations were identified using canonical markers: T cells (CD3^+^), B cells (CD19^+^/B220^+^), dendritic cells (CD11c^+^), neutrophils (LY6G^+^CD16^+^), monocytes (LY6C^+^CD11b^+^), and macrophages (F4/80^+^). Subsets were defined as CD69^+^ or CD44^+^ activated T cells and *Tgfb*^+^ macrophages. NK1.1^+^ NK, CD8^+^, and CD4^+^ T cells were not detected. Dimensionality reduction was performed using opt-tSNE (111), and cell clusters were visualized via scatter, heatmap, and contour overlays. Cell population abundance between groups was compared using violin plots. Gating thresholds were determined from biaxial density plots, with CD45^+^ cell counts matched across conditions.

### Single-cell sequencing and pseudotime analysis.

scRNA-Seq data from the LNR experiment were processed using Seurat v5, excluding cells with <200 genes, >10% mitochondrial reads, >5,500 nFeature-RNA, or >30,000 nCount-RNA. Data integration and batch correction were performed using SCTransform and Harmony, followed by clustering at a resolution of 0.7 and annotation based on canonical marker genes.

### Digit tip amputation datasets.

(P2: 10,14 DPA; P3: 7,10,14,28,56 DPA; NCBI GEO GSE135985) (112), were processed in Seurat v5 (113), using similar quality control thresholds. After normalization and principal component analysis (30 PCs), Harmony integration and clustering (resolution 0.5) were performed, and FeaturePlot visualized uniform manifold approximation and projection gene expression. Marker genes were identified using the Wilcoxon rank-sum test (adjusted *P* < 0.05). For pseudotime trajectory analysis, Monocle v3 (114) was used on myeloid and osteoclast subsets. P2 samples were downsampled (~1,400 cells) to match P3. Trajectories were generated with learn-graph and order-cells, and top markers were determined per branch. Pseudotime analysis revealed that monocytic precursors in both P2 and P3 diverged into dendritic cell–like precursors, consistent with prior findings (68).

### Statistics.

Quantitative data are expressed as a mean ± SD with individual data points shown, unless otherwise stated. *P* < 0.05 was considered significant, and adjustments were not made for multiple comparisons. The number of samples is also indicated in figure legends. Parametric data were analyzed using an appropriate 2-tailed paired Student’s *t* test comparing paired littermate controls or 1-way ANOVA with Tukey’s post hoc analysis for comparison of multiple groups.

### Study approval.

All animal experiments described were approved by the Institutional Animal Care and Use Committee at the University of Texas Southwestern Medical Center. This study was carried out in accordance with the *Guide for the Use and Care of Laboratory Animals* from the Institute for Laboratory Animal Research (National Academies Press, 2011).

### Data availability.

All next-generation sequencing data generated in this study are publicly available through NCBI Gene Expression Database (GEO GSE313923). Supporting datasets, including quantified values and analysis outputs, are provided in XLS format as a Supporting Data Values file.

## Author contributions

NV and BL conceived the study and designed the experiments. NV developed the methods. NV, TKG, MY, and DGW performed the experiments. NV, TKG, MM, and SK analyzed the data. JHLC, SK, EG, and CJ assisted with quantification. JLC assisted with scRNA-seq and LNR experiments. SGC, BM, and DAB provided scientific input. NV prepared the manuscript. NV, BL, LAD, and MTD edited the manuscript. All authors reviewed and approved the final manuscript.

## Funding support

- NV by the CRSM award from UTSW.

## Supplementary Material

Supplemental data

Supporting data values

## Version 1 02/02/2026

Electronic publication
