# Supplementary material for: Targeting lymphatic vessels enhances bone regeneration by augmenting osteoclast activity in mouse models of amputation
Source: J Clin Invest. 2026 Feb 2;136(3):e191906. doi: 10.1172/JCI191906 (PMC12867134; doi:10.1172/JCI191906)
Supplement: Supplemental data [file jci-136-191906-s092.pdf]

Supplementary figures and tables:

Figure S1. Dynamic pattern of lymphangiogenesis during mouse digit tip regeneration.

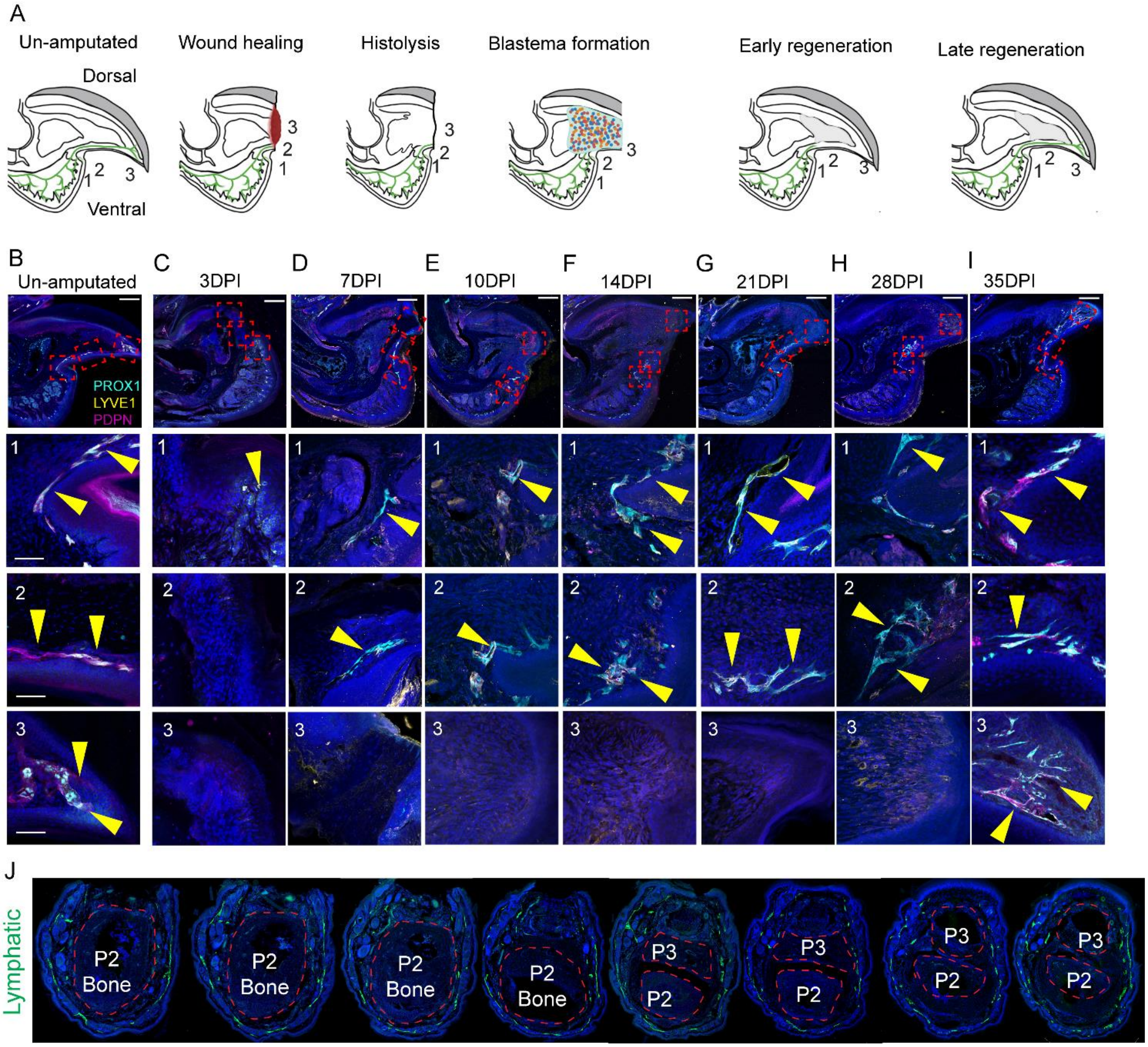

(A) Schematic illustration of lymphatic vessel distribution in the unamputated digit and throughout the regenerative stages following distal amputation. Green lines indicate lymphatic vessels, and numbers (1–3) correspond to regions along the ventral digit analyzed in panels B–I. **(B–I)** Representative longitudinal sections from *Prox1-eGFP* reporter digits immunostained for lymphatic markers LYVE1 (Yellow) and PDPN (Magenta) and counterstained with DAPI (blue) at indicated days post-injury (DPI). **(B)** Unamputated digits show lymphatic vessels confined to the ventral dermis. **(C–E)** By 3–10 DPI, lymphatic sprouts emerge at the ventral wound margin (arrowheads). **(F–H)** During the early and mid-regenerative phases (14–28 DPI), lymphatic vessels expand and remodel around the regenerating mesenchyme and near the bone stump, remaining restricted to soft tissue. **(I)** At 35 DPI, a mature, organized lymphatic network is re-established along the ventral side of the regenerated digit. Magnified insets (rows 1–3) correspond to the numbered ventral regions in schematic (A); yellow arrowheads highlight PROX1+/LYVE1^{+}/PDPN^{+} lymphatic structures. Scale bars: upper panels, 200 µm; lower panels, 100 µm. **(J)** Transverse cross-sections through unamputated digit showing lymphatic vessels (PROX1+/LYVE1^{+}/PDPN^{+}) within the ventral side of digit. Some of the images in this figure also appear in Figure 1. To visualize the entire ventral region, certain magnified panels include overlapping areas from adjacent fields

Figure S2. **Osteoclast differentiation and activity during regenerative versus non-regenerative digit healing.**

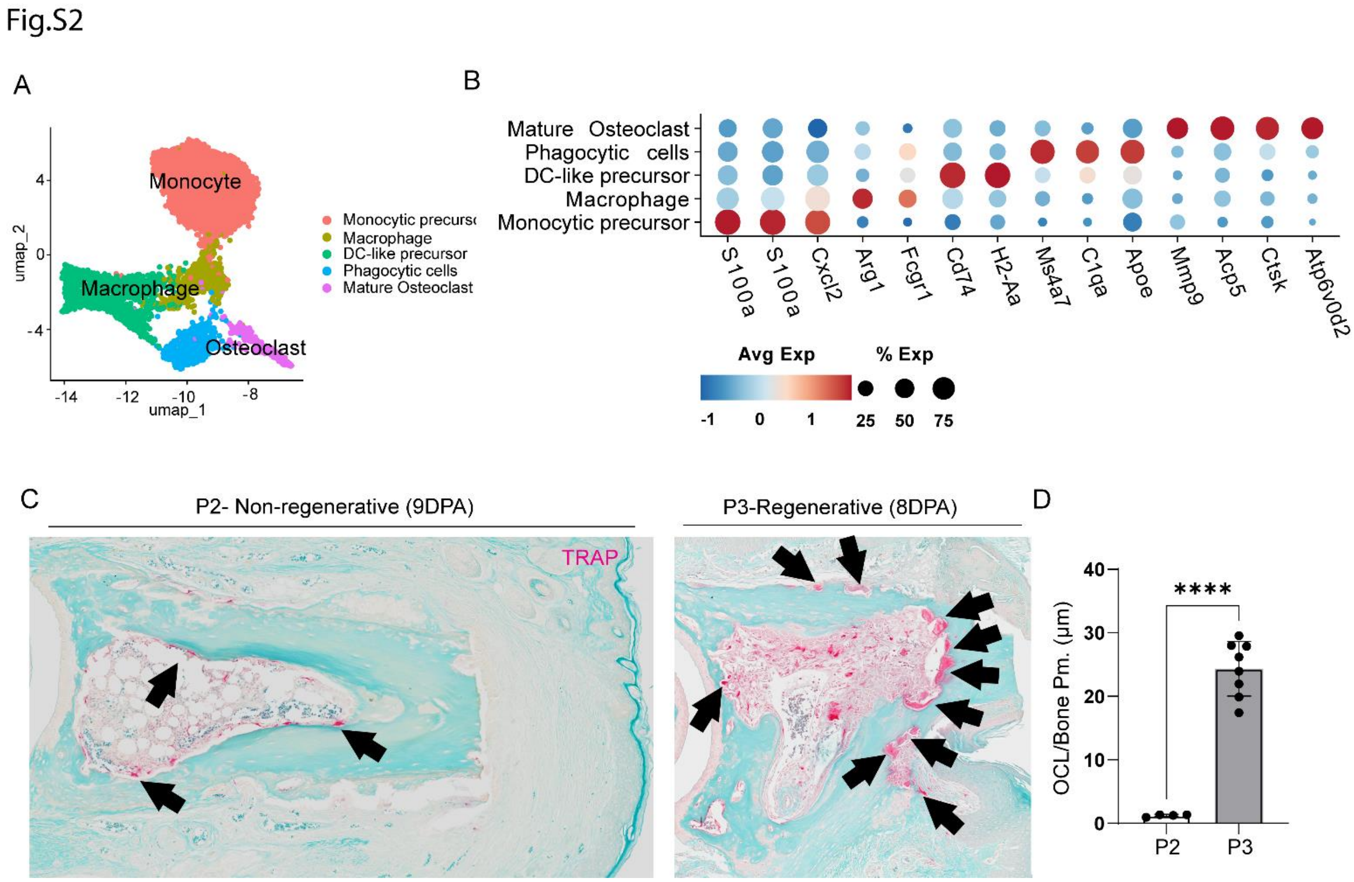

(A) UMAP of myeloid lineage clusters from single-cell RNA-seq of regenerating digit tissue identifies distinct populations of monocytic precursors, macrophages, dendritic-like precursors, phagocytic cells, and mature osteoclasts. **(B)** Dot plot shows unique gene expression across identified clusters. **(C)** TRAP staining of longitudinal sections from non-regenerative (P2) and regenerative (P3) digits at 8–9 DPA. **(D)** Quantification of osteoclast surface per bone perimeter (OCL/Bone Pm) demonstrates a significant increase in osteoclast activity in regenerating P3 digits compared with non-regenerative P2 digits (**** $P < 0.0001$, unpaired two-tailed t-test).

Figure S3. Inhibition of VEGFR3 increases osteoid formation during digit tip regeneration.

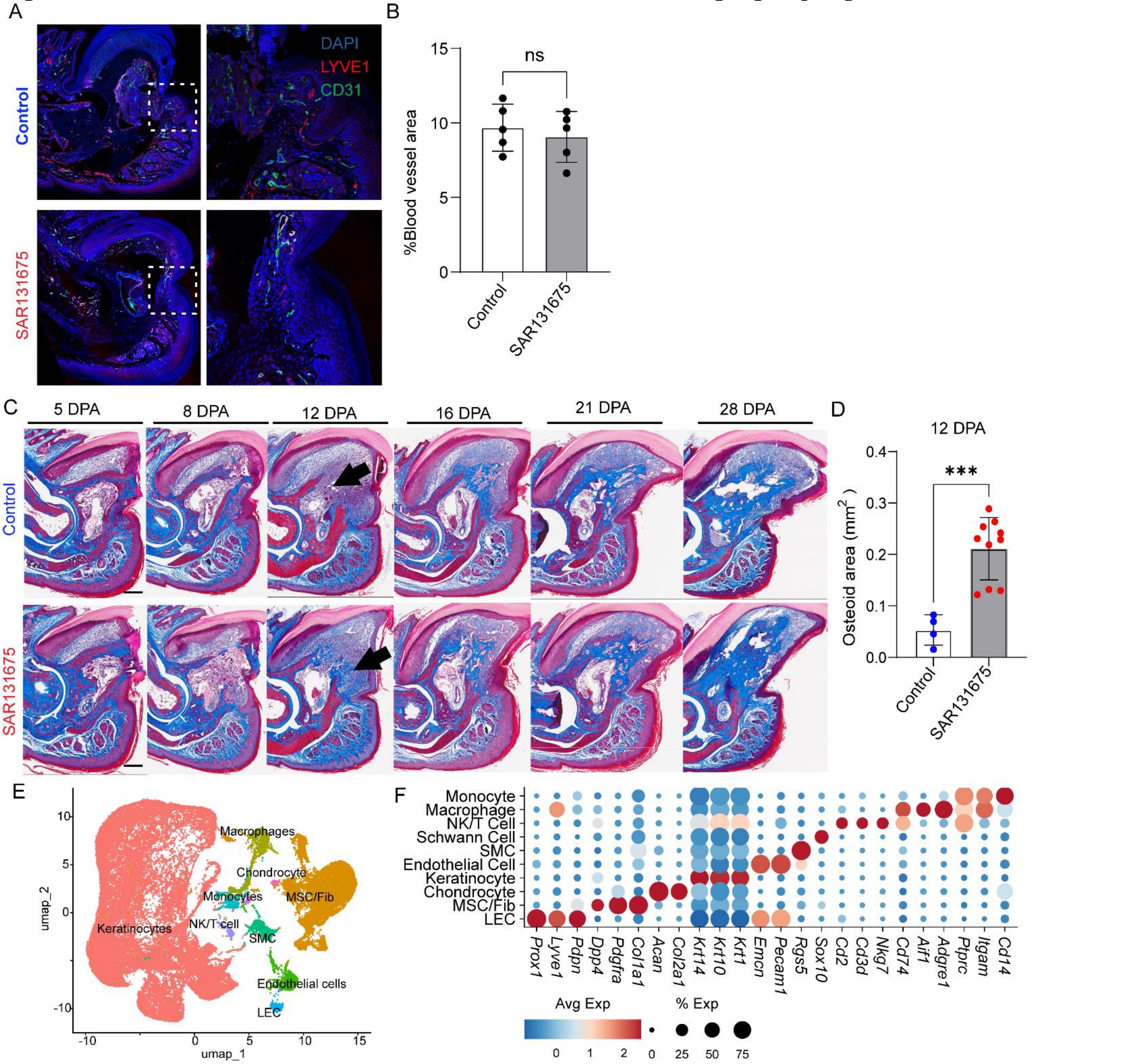

(A) Immunofluorescent staining for LYVE1 (red) and CD31 (green) in control and SAR131675-treated digits at 8 DPA. **(B)** Quantification of $CD31^+$ blood vessel area at 8 DPA shows no significant difference between control and SAR131675-treated groups, indicating that VEGFR3 inhibition selectively affects lymphatic, not blood, vasculature. Data represent mean ± SD; Student's t test; ns, not significant. **(C)** Representative longitudinal sections stained with Masson's Trichrome, at multiple time points (5–28 DPA) in control and SAR131675-treated digits. Arrows highlight regions of prominent osteoid deposition at 12 DPA. Scale bars, 200 µm. **(D)** Quantification of osteoid area at 12 DPA demonstrates a significant increase in SAR131675-treated digits compared with controls, suggesting that inhibition of lymphangiogenesis promotes early osteoid formation. Data represent mean ± SD; Student's t test; *** $p < 0.001$. **(E)** UMAP visualization of single-cell RNA-seq data from regenerating digit tissue at 8DPA. **(F)** Dot plot showing cell type-specific expression of unique genes across major clusters.

Figure S4. Gating strategies used for CyTOF analysis of immune cells from mice fed with the VEGFR3 inhibitor SAR131675 or a control diet.

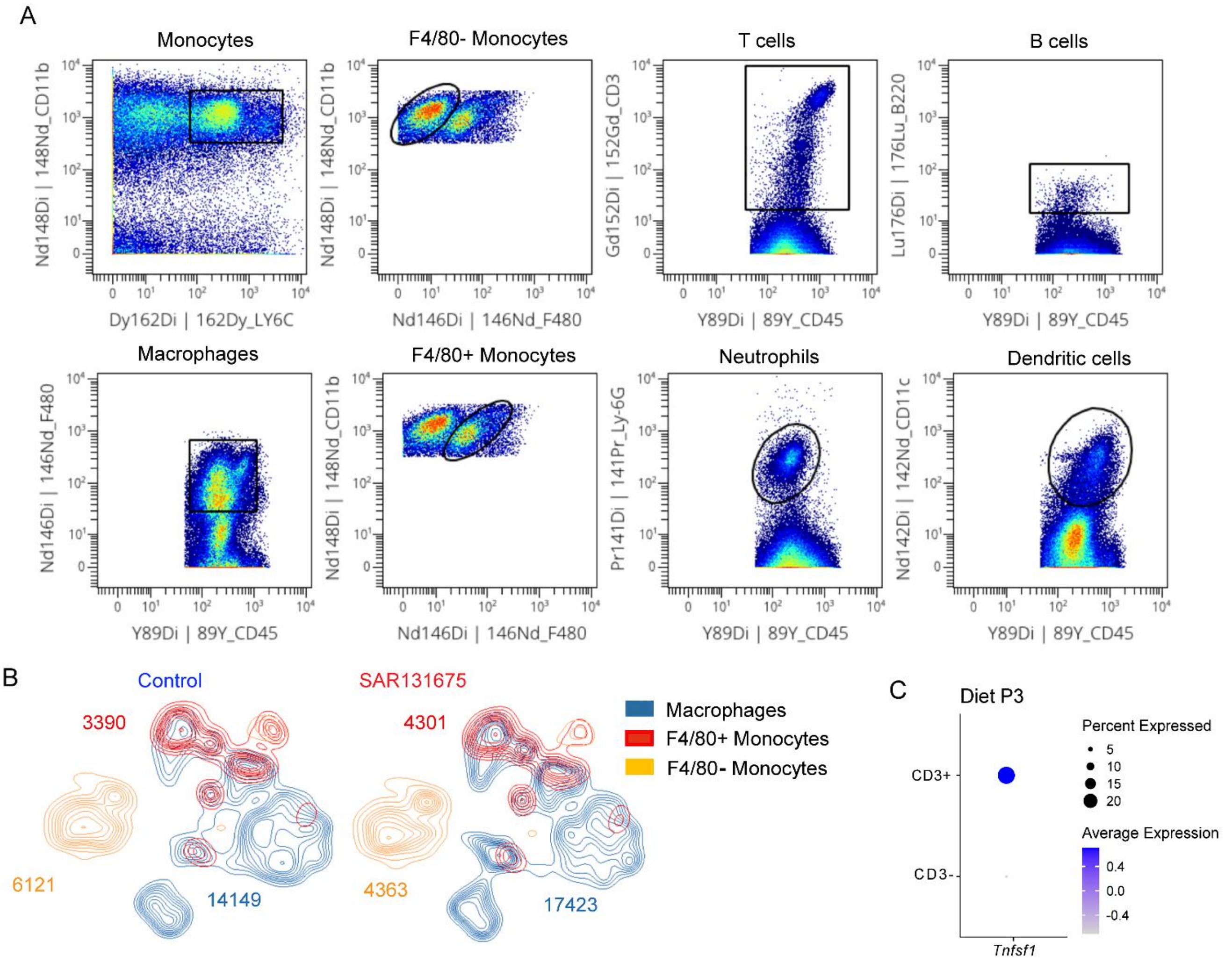

(A) Representative gating strategy used to identify major immune cell populations in regenerating digit tissue at 5 DPA. Monocytes were gated as $CD11b^{+}Ly6C^{+}$ cells, macrophages as $CD45^{+}F4/80^{+}$ cells, and neutrophils as $CD45^{+}Ly6G^{+}CD11c^{-}$ cells. Dendritic cells were defined as $CD45^{+}CD11c^{+}$, T-cells as $CD45^{+}CD3^{+}$, and B cells as $CD45^{+}B220^{+}$. Subgating of $F4/80^{+}$ populations distinguished $F4/80^{+}$ monocytes and F4/80- monocytes for downstream analysis. **(B)** SPADE maps comparing control (blue) and SAR131675-treated (red) digits highlight increased frequencies of macrophages and $F4/80^{+}$ monocyte subsets following VEGFR3 inhibition. Numbers indicate relative cell counts for each cluster. **(C)** Dot plot showing Tnfsf1 expression (encoding TNF ligand superfamily member 1) within $CD3^{+}$ T-cells versus $CD3^{-}$ populations from diet-treated digits, illustrating upregulation of Tnfsf1 in the T-cell compartment.

Figure S5. Osteoid formation accelerated after LEC ablation.

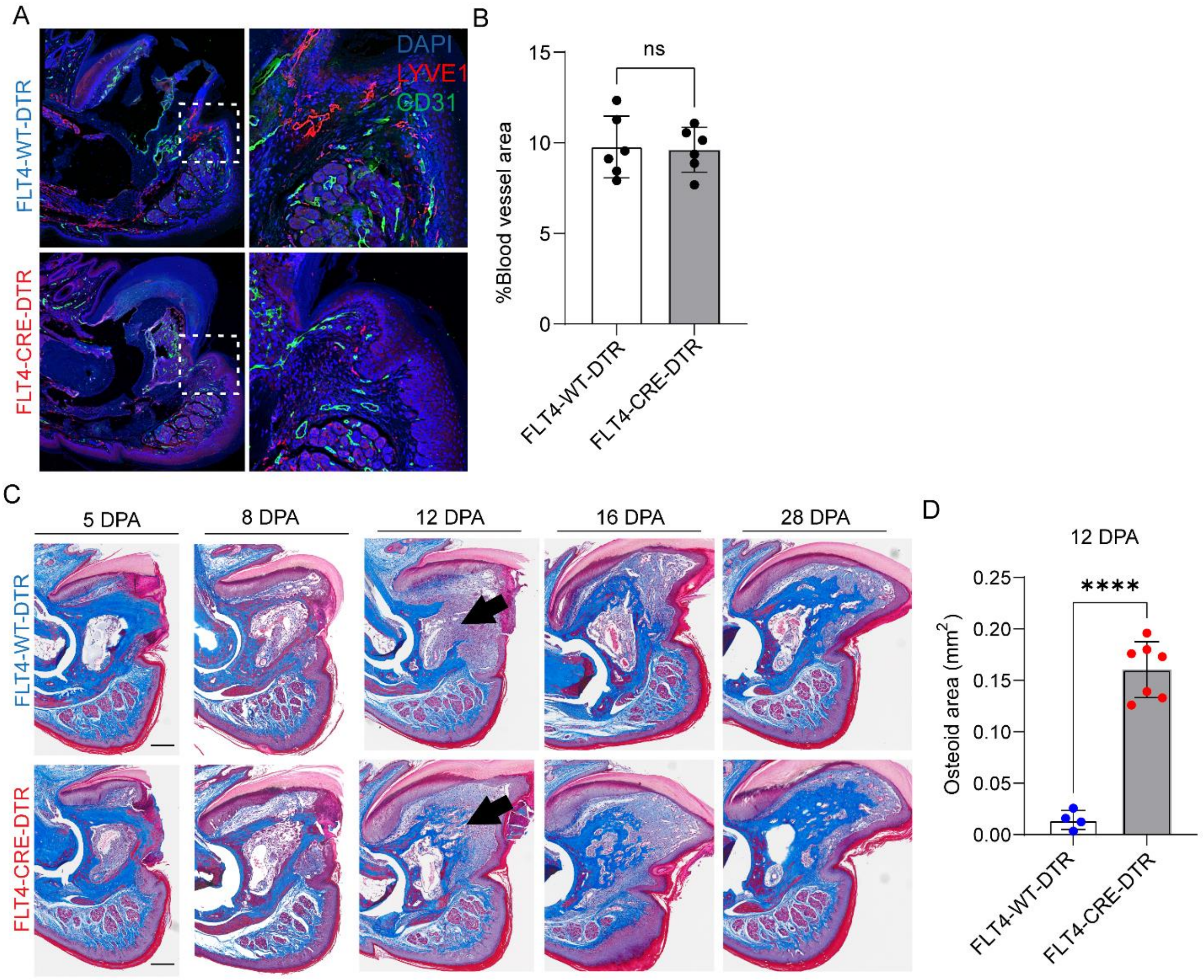

(A) Immunofluorescent staining for blood vessel marker CD31 (green) and lymphatic marker LYVE1 (red) in control and *Flt4Cre*$^{ERT2+}$*; iDTR+* digits at 8 DPA shows intact blood vasculature in both genotypes. **(B)** Quantification of blood vessel area reveals no significant difference between control and *Flt4Cre*$^{ERT2+}$*; iDTR+* digits. **(C)** Representative Trichrome-stained longitudinal digit sections at indicated time points (5, 8, 12, 16, and 28 DPA) showing increased osteoid deposition (black arrows) in *Flt4Cre*$^{ERT2+}$*; iDTR+* digits compared to controls at 12 DPA. Scale bar: 200 µm. **(D)** Quantification of osteoid area at 12 DPA shows a significant increase in *Flt4Cre*$^{ERT2+}$*; iDTR+* digits relative to controls (mean ± SD; ****$p < 0.0001$, Student's t test).

Figure S6. Impact of genetic *Vegfr3* deficiency on osteoclast activity and digit tip regeneration.

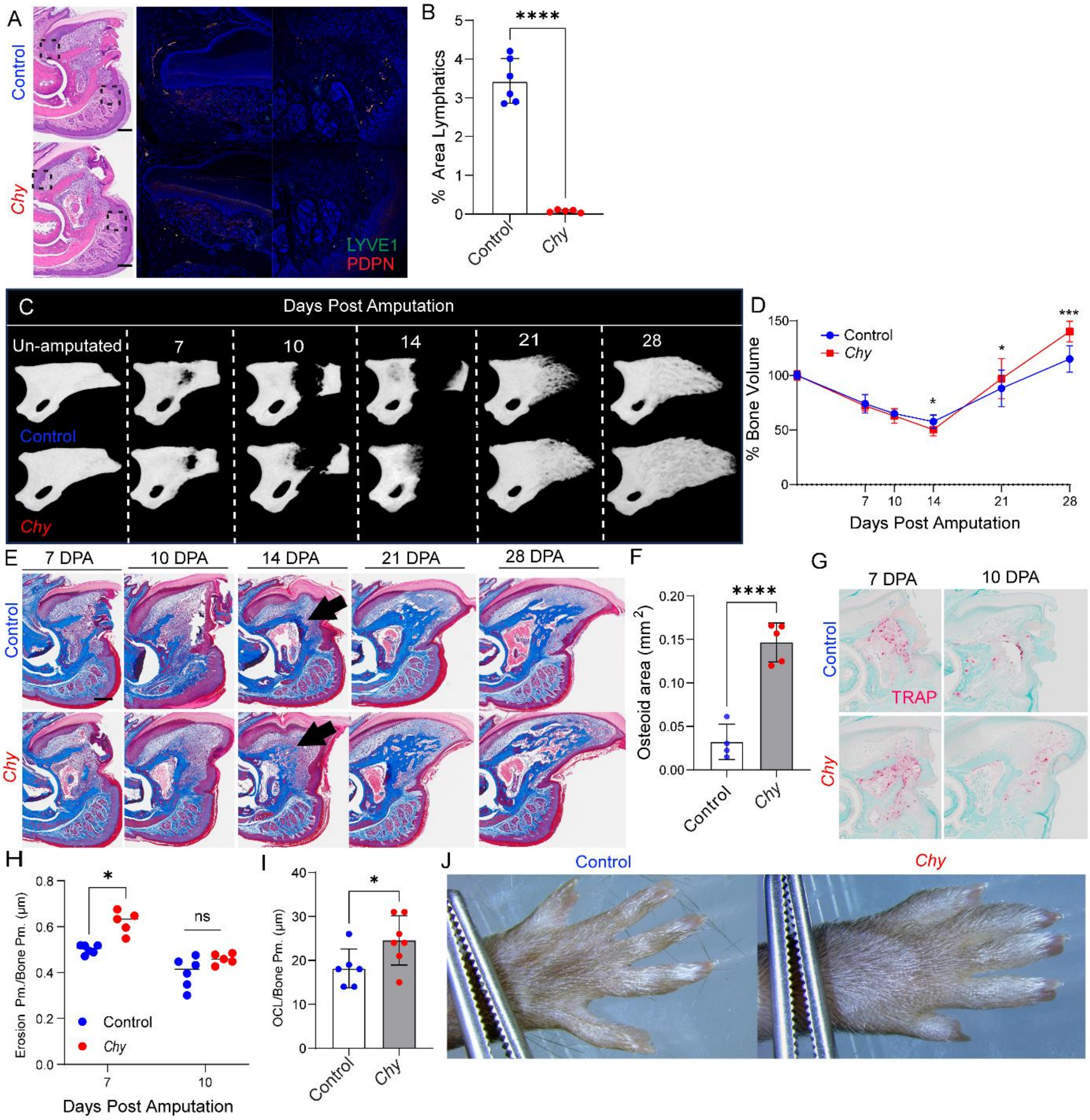

(A) Representative H&E and immunofluorescent images of digits from *Chy* (*Vegfr3*$^{+/-}$) and control mice showing markedly reduced lymphatic vessel formation, as indicated by LYVE1 and PDPN staining. Scale bar: 200 µm. **(B)** Quantification of LYVE1+ lymphatic area confirms a significant reduction in lymphangiogenesis in *Chy* digits compared to controls (mean ± SD; ****p < 0.0001, Student's *t* test). **(C)** Representative longitudinal µCT images showing regenerative progression in control and *Chy* digits at the indicated days post-amputation. **(D)** Quantification of bone volume reveals a transient acceleration in bone resorption in *Chy* mice during early stages, followed by accelerated bone regrowth at later stages (mean ± SD; *p < 0.05, ***p < 0.001). **(E)** Representative Trichrome-stained longitudinal digit sections showing increased osteoid deposition (Black Arrow) in *Chy* digits compared to controls, especially at 14 DPA. Scale bar: 200 µm. **(F)** Quantification of osteoid area at 14 DPA demonstrates a significant increase in *Chy* digits (****p < 0.0001). **(G)** TRAP-stained sections at 7 and 10 DPA showing enhanced osteoclast activity in *Chy* digits. **(H–I)** Quantification of osteoclast-mediated erosion perimeter (H) and osteoclast number per bone perimeter (I) showing increased osteoclastic activity in *Chy* digits during early regeneration (mean ± SD; *p < 0.05). **(J)** Bright-field images of whole hind paws showing normal appearance in control mice and lymphedematous morphology in *Chy* mice.

Figure S7. Lymph node removal enhances osteoid formation and accelerates bone regeneration through osteoclast activation.

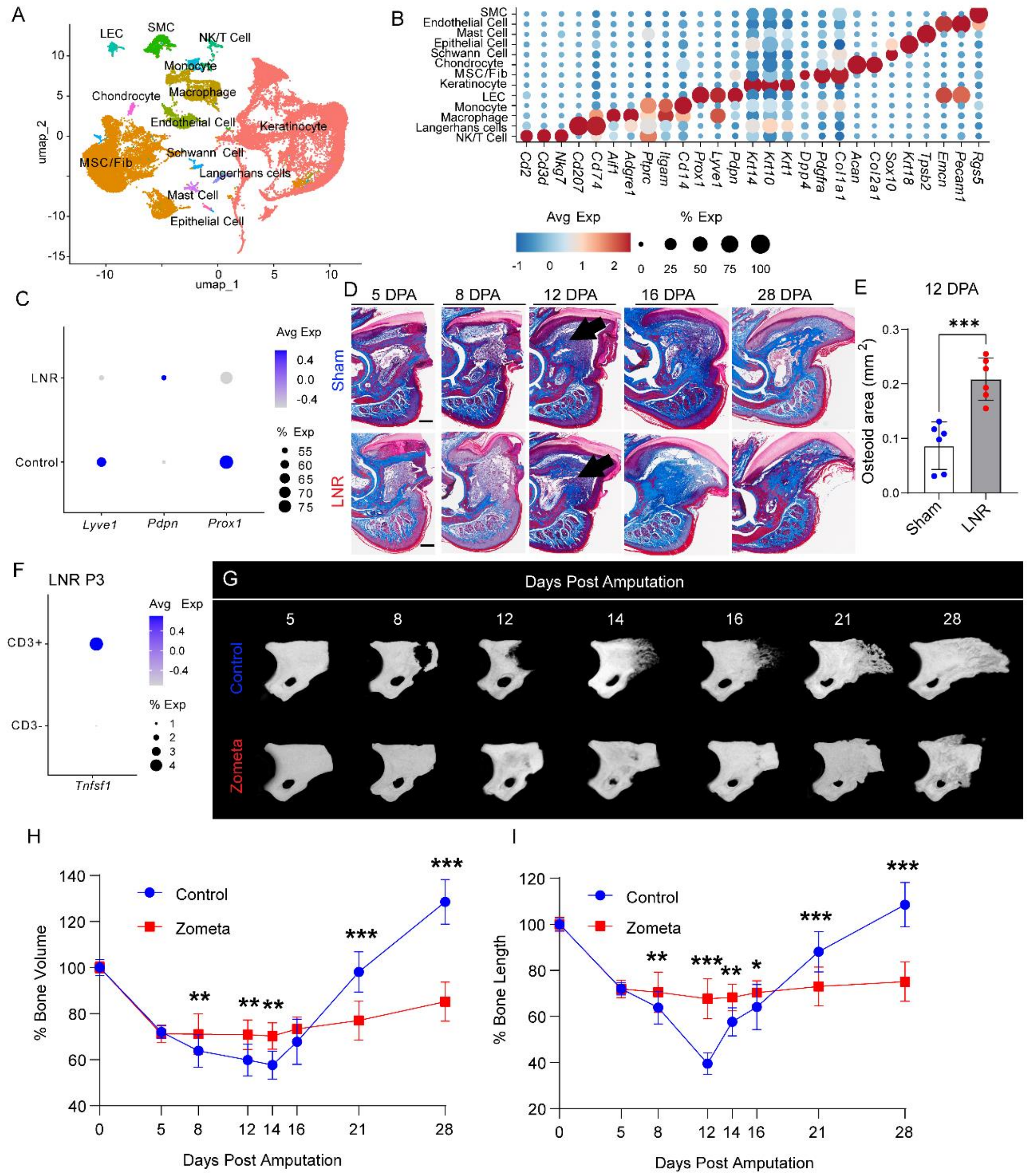

(A) UMAP visualization of single-cell transcriptomic data showing distinct cellular clusters. **(B)** Dot plot showing expression of selected marker genes for major identified cell types. **(C)** Dot plot comparing the expression of lymphatic markers (*Lyve1*, *Pdpn*, *Prox1*) between LNR and control digits, confirming reduced lymphatic signature following lymph node removal. **(D)** Trichrome staining of longitudinal digit sections from sham and LNR mice at 5, 8, 12, 16, and 28 DPA, showing increased osteoid formation in LNR. **(E)** Quantification of osteoid area confirms a significant increase in bone matrix deposition in LNR digits compared with sham control digits at 12 DPA (**p < 0.001). **(F)** Dot plot showing increased *Tnfsf11* (RANKL) expression in CD3^{+} T-cells in LNR P3 digits at 8 DPA. **(G)** Representative longitudinal micro-CT reconstructions of control and Zometa-treated digits at 5–28 DPA showing impaired bone remodeling after osteoclast inhibition. **(H-I)** Quantification of bone volume **(H)** and bone length **(I)** over the regenerative timeline demonstrates that Zometa treatment significantly reduced bone resorption and delayed bone regrowth (**p < 0.01, **p < 0.001; n = 6–8 digits/group; mean ± SD; Student's t test).

Table S1. Top 30 genes used to calculate LEC and osteoclast scores.

Score	Genes	
LECs	Cldn5	Sdpr
	Mmrn1	Podxl
	Ccl21a	Clic5
	Lyve1	Pard6g
	Flt4	Galnt18
	Prox1	Ttc9
	Reln	Icam2
	Ntn1	Kank3
	Nts	Prelp
	Pcsk6	Lbp
	Sema3d	Palm
	Sh3gl3	Map4k2
	Slc45a3	Fxyd6
	Gpm6a	Pvrl2
	Dmtn	Clu
Osteoclasts	Acp5	Shtn1
	Gpr137b	Matk
	Ccl9	Tnfrsf11a
	Slc37a2	Adrbk2
	Csf1r	Ocstamp
	Clec4a2	Mfsd12
	Atp6v0d2	Cyp2s1
	Atp6v1b2	Fam195a
	Tcirg1	Tfrc
	Pstpip1	Nudt22
	Bdh2	Gnptab
	Chchd10	Arsb
	Mmp9	Snx10
	Slc9b2	Ctsk
	Clec12a	Ckb
